# Morphological Changes in PEDOT:PSS under Electrolytes, Dopamine, and PEG-400 Exposure: A Molecular Simulation Perspective

**DOI:** 10.1021/acs.macromol.5c02727

**Published:** 2026-01-27

**Authors:** Amali G. Guruge, Hesam Makki, Alessandro Troisi

**Affiliations:** Department of Chemistry, 4591 University of Liverpool, Liverpool L69 3BX, U.K.

## Abstract

Poly­(3,4-ethylenedioxythiophene):poly­(styrene sulfonate) (PEDOT:PSS) is a widely used conducting polymer, whose conductivity can be enhanced by incorporation of specific chemical components, whereas diffusion of water into the material can reduce its conductivity. These changes are typically linked to morphological changes in lamella crystallite size, π–π stacking, chain orientation, and interlamella connectivity. However, an atomistic-level understanding of how specific chemical components influence these properties remains limited, particularly in relation to experimentally observed conductivity trends. In this study, molecular dynamics (MD) simulations are employed to investigate the effects of electrolytes, dopamine, and poly­(ethylene glycol) 400 (PEG-400) on PEDOT:PSS morphology and relate the findings to experimental observations. All chemical components were found to screen electrostatic interactions between PEDOT and PSS, potentially affecting the conductivity. Dopamine tends to reduce conductivity by intercalating between PEDOT and PSS, disrupting interdomain connectivity. In contrast, PEG-400 enhances conductivity by improving interlamellar connectivity without altering PEDOT chain conformation, challenging conventional explanations and suggesting an alternative mechanism. CuCl_2_ enhances conductivity via PEDOT conformational changes associated with partial PSS loss, whereas NaCl shows minimal morphological changes, in agreement with established explanations. Overall, MD simulations confirm the established trends, provide alternative insights, and challenge commonly accepted explanations, demonstrating their utility in validating, refining, and reinterpreting molecular mechanisms in complex polymer systems.

## Graphical abstract

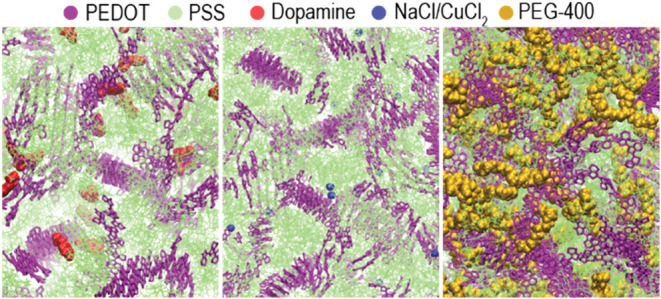

## 1 Introduction

Poly­(3,4-ethylenedioxythiophene):poly­(styrene sulfonate) (PEDOT:PSS) is a widely studied conducting polymer consisting of positively charged PEDOT chains derived from repeating 3,4-ethylenedioxythiophene (EDOT) units and negatively charged PSS chains derived from repeating styrene sulfonate (SS) units. The positively charged PEDOT and negatively charged PSS chains are held together by electrostatic interactions. As a conjugated polymer with alternating single and double bonds, PEDOT enables π-electron delocalization, facilitating electronic conductivity. PEDOT:PSS is highly valued in bioelectronics,^1,2^ flexible electronics,^3^ and energy storage^4^ applications due to its tunable conductivity,^5,6^ thermal stability,^7^ optical transparency,^8–10^ and biocompatibility.^11,12^ It is a key material in organic electrochemical transistors (OECTs), particularly in biosensing.^13,14^ In recent years, molecular dynamics (MD) simulations have become a powerful complementary tool for probing the nanoscale morphology and dynamics of PEDOT:PSS. Prior MD studies have explored the two-phase morphology,^15–17^ its behavior in water,^15,18,19^ and the effects of solvents,^20–22^ ions,^23^ ionic liquids,^24–26^ and pH.^17^ Since the mechanical and electrical properties of PEDOT:PSS are strongly tied to its morphology,^27,28^ affected by PEDOT/PSS composition, synthesis and post-treatment methods, solvents, substrates, and pH conditions,^15^ MD is well suited to investigate the complex structure–property relationships in detail.

One particularly notable property of PEDOT:PSS is its high sensitivity to additives, which can significantly influence its morphology and conductivity. Various additives, including acids,^29,30^ solvents,^20,31,32^ ionic liquids,^6,33–35^ and other compounds,^36–38^ have been shown to enhance conductivity through mechanisms such as improved alignment of PEDOT lamellae,^30,33^ altered interlamellar distances,^29^ modified π–π stacking distances,^33–35^ increased crystallite size,^21,29^ and enhanced phase separation due to charge screening.^20^ Conversely, water diffusion can modify the PEDOT:PSS morphology, potentially reducing the conductivity by decreasing the size and alignment of lamella crystallites and increasing the interlamellar distances.^19^ However, the effects of additional chemical components present in water, particularly electrolytes and biomolecules containing electrolytes, on PEDOT:PSS morphology remain poorly understood. While MD simulations have probed some of these effects,^25^ comprehensive and methodical studies linking conductivity-relevant morphological parameters to specific chemical constituents are still limited.^19,21^ This paper addresses this gap by systematically investigating the influence of three chemical systems: aqueous solutions of NaCl and CuCl_2_, the biomolecule dopamine, and the polar solvent PEG-400, on key morphological parameters related to conductivity. Each system is highly relevant to PEDOT:PSS-based bioelectronics as discussed below.

In OECTs, the PEDOT:PSS interfaces with electrolytes, often containing biomolecules, to transduce ionic signals into electronic ones.^39^ This has driven extensive research into how electrolytes impact the PEDOT:PSS structure and function. Experimentally, ion mobility,^40^ salt-induced conductivity changes,^36,37^ ionic-to-electronic coupling efficiency,^41^ and the effect of ionic strength on morphology^18^ have been studied. MD studies have similarly explored ion diffusion^23^ and changes in morphology, particle size, and zeta potential with ionic strength.^18^ Some studies reported that certain salts enhance conductivity by promoting PSS loss from the film,^36,37^ underscoring the electrolyte’s role in modulating PEDOT:PSS morphology and consequently, its conductivity. Given that bioelectronic devices often contain electrolytes, where ion diffusion could influence the device efficiency, theoretical insights into how specific electrolytes alter conductivity-relevant structural features remain lacking.

Biomolecules in electrolytes can also affect the PEDOT:PSS morphology, especially in biosensing OECTs, where direct material contact occurs. PEDOT:PSS sensors have been used to detect glucose,^42^ uric acid,^43^ lactate,^44^ cholesterol,^44^ and dopamine (DA).^45–47^ DA, a catecholamine neurotransmitter,^48^ was selected for this study due to its role in cognition, learning, reward, and motor control,^49^ with abnormal levels associated with Parkinson’s disease,^50^ autism,^51^ and schizophrenia.^52^ DA detection is crucial for clinical diagnosis and treatment monitoring. In OECT-based sensors, DA is typically detected via electro-oxidation at the gate electrode, generating a Faradaic current that alters the gate/electrolyte potential and modulates the drain current.^45,47^ During this process, DA in electrolytes may also diffuse into PEDOT:PSS, potentially impacting its morphology and sensor performance. Despite this relevance, no MD studies have directly examined how DA influences PEDOT:PSS structural features linked to the conductivity.

Polar solvents like poly­(ethylene glycol)­s (PEGs) are widely used to enhance PEDOT:PSS conductivity.^32,53–57^ Enhancements have been attributed to PEG-induced phase separation between PEDOT and PSS,^54,55,57,58^ rearrangement of PEDOTs into larger, more connected particles,^55^ and reduced π–π stacking distances.^56^ However, the collective contribution of conductivity-related structural properties to the observed enhancements remains unclear. Specifically, it is not yet known whether PEG induces multiple alterations in conductivity-related morphological properties or only one or two changes that could account for experimentally observed conductivity enhancement. Among various PEGs known to enhance conductivity (e.g., PEG-200,^55,57^ PEG-400,^55–58^ PEG-600 ^55,57^ ), PEG-400 was selected as a representative for this study.

In summary, this study investigates how aqueous solutions of NaCl and CuCl_2_, dopamine, and PEG-400, modulate key structural parameters known to influence PEDOT:PSS conductivity. In our systems, PEG-400 functions as an additive, whereas NaCl, CuCl_2_, and dopamine are studied in terms of their effects upon diffusing into the material. By combining systematic MD simulations with experimental correlations, we aim to provide molecular-level insights into the mechanisms governing PEDOT:PSS performance in bioelectronic applications.

## 2 Methods

Since PEDOT:PSS thin films comprise conductive PEDOT-rich and -less conductive PSS-rich regions,^59^ our models included both phases and reflected experimentally observed PEDOT concentrations (see Table 1 for details).^60^ In the phase structures, PEDOT was modeled using oligomers composed of 12 EDOT repeating units in the bipolaronic state (+4) (Figure 1a). This choice ensures that PEDOT reflects an oxidation level of 33% (i.e., one positive charge per three monomer units), consistent with values commonly reported in the literature for PEDOT chains.^61,62^ PSS chains consisted of 35 repeating units, incorporating either four or eight SS– monomers and SSH monomers (Figure 1b,e), randomly distributed along the chain. The random placement ensured a statistically independent mixture of PSS chains with various repeating unit sequences.^17^ The number of PEDOT and PSS chains was chosen to maintain the overall charge neutrality in each system.

**Figure 1:**
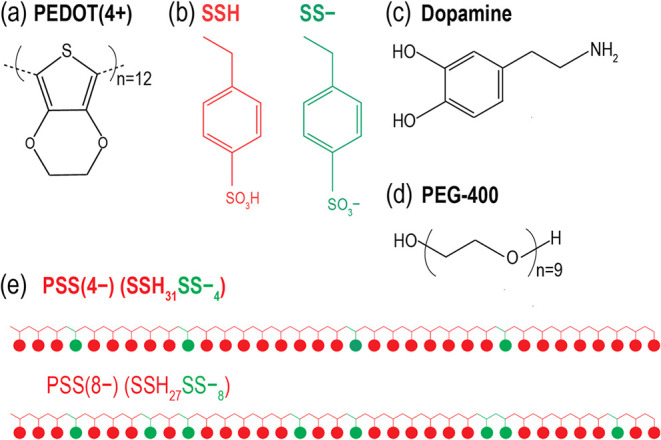
(a) Chemical structure of PEDOT. (b) Chemical structures of the repeat units of PSS in the SSH and SS- forms. (c) Chemical structure of dopamine. (d) Chemical structure of PEG-400. (e) PSS model used in this study, consisting of 35 repeat units per chain, with either 4 or 8 randomly distributed SS– monomers and 31 or 27 SSH monomers, respectively..^16^

**Table 1:** Composition of Molecular Components in the Simulated PEDOT-Rich and PSS-Rich Phases

Molecular details	PEDOT-rich	PSS-rich
PEDOT wt % in PEDOT:PSS	52	26
PEDOT chains	120	60
PSS(-4)	0	60
PSS(-8)	60	0
no. of Na^+^ ions^a^	16	16
no. of Cu^2+^ ions^a^	16	16
no. of dopamine^a^	26	26
PEG-400 (4% v/v)^a^	50	42
PEG-400 (10% v/v)^a^	124	105

a Only one chemical component (i.e., Na^+^, Cu^2+^, dopamine, or PEG-400) was introduced into each of the PEDOT-rich and PSS-rich systems.

### 2.1 Construction of Systems with PEDOT:PSS–Electrolytes, Dopamine, and PEG-400

To construct PEDOT:PSS systems containing electrolytes and dopamine (Figure 1c), we used well-equilibrated water-diffused PEDOT-rich and PSS-rich phase structures from our previous study.^19^ The equilibration of these structures used the same force field and annealing procedures employed in the current study. Each structure included both a bulk-water region and a water-channel region, as illustrated in Figure 2a. In dopamine and electrolyte systems, the PEDOT-rich and PSS-rich structures contained 15070 and 12,860 water molecules, respectively. The concentrations of ions were set to ∼0.1 mol dm^–3^, while the dopamine concentration was set to ∼0.2 mol dm^–3^ to ensure a sufficient number of ions/molecules within the MD simulation system. The number of Cl^–^ ions was adjusted to neutralize the total positive charge introduced by the Na^+^ and Cu^2+^ ions. Depending on the system, ions or dopamine molecules were randomly inserted into the water-channel region of the structure (see Table 1), ensuring no overlap with existing atoms using the Silico package.^63^ Placing ions or dopamine directly within the water-channel region ensures that a sufficient number of these species are present in the bulk polymer, thereby avoiding significantly longer simulations that would be required if they were initially placed in the bulk-water regions. Moreover, over the time scale of our simulations, both ions and dopamine remain stably confined within the water-channel region, with no measurable depletion or spontaneous migration into the bulk-water phase, providing confidence that these chemical components are well equilibrated within the bulk polymer region. A representative initial structure of the PSS-rich phase with dopamine is shown in Figure 2a.

**Figure 2:**
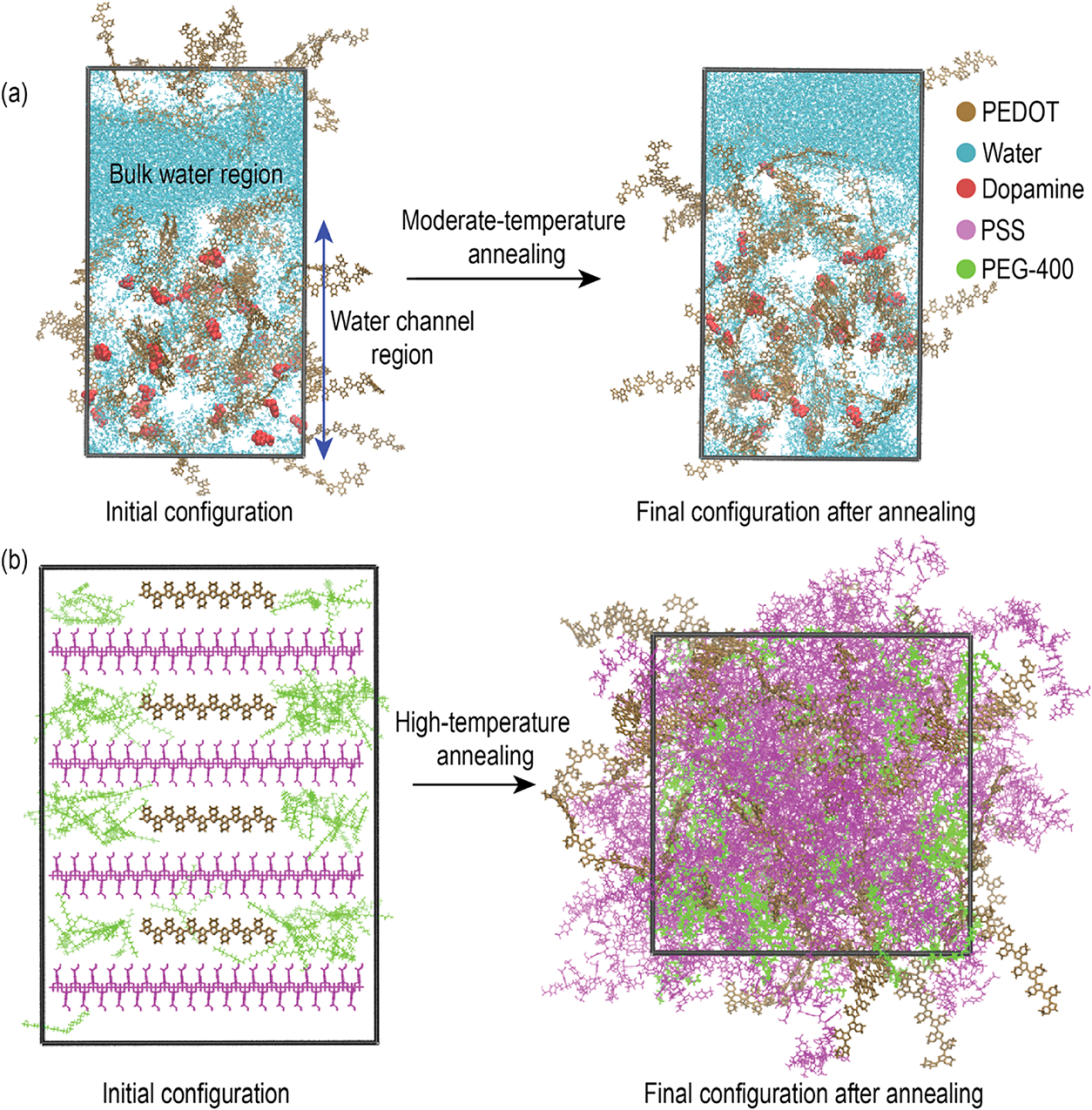
MD simulation protocols followed for (a) systems containing ions or dopamine and (b) systems containing PEG-400. Representative images shown are for the PSS-rich phase containing dopamine and 4% v/v PEG-400.

To build the PEDOT:PSS-PEG-400 systems, PEDOT-rich and PSS-rich phases were assembled by stacking 15 layers of PEDOT chains, as specified in Table 1 onto a PSS template.^16^ PEG-400 molecules (Figure 1d) were then randomly inserted into the system to achieve 4 and 10% v/v concentrations, as conductivity enhancement has been reported at similar concentrations.^55^ A representative initial configuration of the PSS-rich phase with 4% v/v PEG-400 is shown in Figure 2b.

### 2.2 MD Simulations

All simulations were performed using GROMACS,^64,65^ version 2022.0. Water molecules were modeled using the SPC/E model. The Generalized Amber Force Field (GAFF)^66^ was used to model the PSS chains. For PEDOT, we employed recently developed and validated all-atom force-field parameters^67,68^ that are compatible with the GAFF. These parameters accurately reproduce key morphological features, such as the π–π stacking distance and the average size of PEDOT lamella crystallites,^16,69^ in agreement with experimental observations. This consistency provides confidence in the adequacy of the force field to model the charge transport behavior of PEDOT:PSS and to predict conductivity-related properties in this study. GAFF-compatible all-atom parameters for PEG-400 and dopamine were generated using the PolyParGen^70^ server. Classical force fields typically assign consistent atomic charges to chemically equivalent atoms in PEG systems.^71,72^ However, the atomic charges assigned to PEG-400 by PolyParGen showed slight inconsistencies among chemically equivalent atoms (Table S1). To address this, we modified the charges to ensure consistency across chemically equivalent atoms using the density functional theory (DFT) approach (see Section S1 in the Supporting Information, SI, for details). Since DFT-derived charges are not automatically the best choice for PEG-400 in our systems, we then evaluated both the modified charge model (i.e., PEG-400 with consistent charges) and the original PolyParGen-assigned model by comparing their ability to reproduce known physical properties of PEG-400 (see Section S2I in the SI for details). As shown in Table S2 in the SI, the calculated density, radius of gyration (*R*_g_), and end-to-end distance using the modified charges were in good agreement with values reported in the literature. The dopamine parameters generated by PolyParGen were also validated by comparing its diffusion coefficient in water with experimental data (Section S2II in the SI). The calculated value showed good agreement with the experimental data (Table S3 in the SI). Ions were modeled as follows: Na^+^, Cl^–^, and Cu^2+^ were modeled using Lennard-Jones (LJ) parameters from Joung and Cheatham^73^ and Babu and Lim,^74^ which were developed for use with the AMBER force field. The compatibility of ion parameters with the GAFF force field has been demonstarted in previous studies, providing confidence for their use in the present work.^69,75^ In addition, we further validated these parameters by performing additional tests of ion diffusion. For example, a simulation box containing CuCl_2_ was modeled to evaluate whether the diffusion of Cu^2+^ and Cl^–^ ions is accurately captured by the chosen parameters (see Section S2II in the SI). The good agreement between the calculated and experimental diffusion coefficients for the ions (see Table S3) provided confidence to proceed with the study using the selected LJ parameters. Simulations used a 2 fs time step, with all H-bonds constrained. Temperature coupling was performed using the velocity rescaling^76^ thermostat, and pressure coupling was applied isotopically using the Berendsen^77^ barostat during the initial equilibration and the Parrinello–Rahman^78^ barostat during simulated annealing. Pressure was maintained at 1 bar, with a compressibility of 4.5 × 10^–5^ bar^–1^. Short-range nonbonded interactions were treated using the Verlet cutoff scheme^79^ with a 1.4 nm cutoff, while long-range electrostatic interactions were calculated using the particle-mesh Ewald^80^ (PME) method with a real-space cutoff of 1.4 nm and a grid spacing of 0.12 nm.

After initial equilibration, production simulations were performed to ensure full relaxation of the systems. Given the high-glass-transition temperature (*T*_g_) of PEDOT:PSS (∼1050 K^81^) observed in simulations, high-temperature annealing is typically required to achieve faster equilibration. However, in systems containing water, elevated temperatures can induce significant volume fluctuations, which may hinder the equilibration process.^19^ To address this, two annealing protocols were employed based on the system constituents. For water-containing systems (i.e., those with ions and dopamine), an annealing protocol using moderate temperatures (≤360 K) was applied, as detailed in Table S4 of the SI. This protocol, previously validated for equilibrating PEDOT-rich and PSS-rich phases with water, was effective in producing well-equilibrated PEDOT:PSS–water models.^19^ MD simulations using this moderate-temperature annealing protocol were carried out for 1 μs or longer for both dopamine-containing and electrolyte systems. For systems containing PEG-400, a sub-*T*_g_ annealing protocol^81^ was employed, involving temperatures of up to 1100 K (i.e., above *T*_g_), as detailed in Table S4 of the SI. Simulations using this high-temperature annealing protocol were carried out for 1.3 μs.

We performed additional MD simulations in the NPT ensemble using equilibrated systems obtained from the annealing protocols. These simulations were conducted to calculate the radius of gyration, end-to-end distances, pairwise interaction energies, and radial distribution functions, as described later in the study. All simulation parameters were identical to those described above (Section 2.2 MD Simulations), and each simulation was carried out at 300 K for 100 ns.

### 2.3 Simulation Analysis

To assess how different chemical components affect charge transport in PEDOT:PSS, we analyzed the following conductivity-related parameters. Detailed descriptions of the implementation of these analyses are provided in the Supporting Information (Section S3).

I. Lamella crystallite size and the number of π–π stacked pairs—To evaluate changes in PEDOT lamella crystallite (defined as clusters of PEDOT chains exhibiting π–π stacking interactions), we analyzed, for each system, the average number of PEDOT chains per crystallite (crystallite size), as well as the average number of π–π stacked EDOT pairs, following the methodology described in Section S3I of the Supporting Information.
II. Orientation parameter of PEDOT—To evaluate the alignment of PEDOT chains, their orientational order was quantified using the nematic order parameter, calculated from a Q-tensor constructed from chain orientation vectors^82^ derived from sulfur–sulfur segmental vectors along the polymer backbone. Further details are provided in the Supporting Information (Section S3II).
III. Connectivity between lamella crystallites—To assess how chemical components modify PEDOT crystallite connectivity, we calculated the shortest distances (*D*_sp2‑sp2_) between adjacent sp^2^ carbon atoms in two neighboring crystallites (see the illustration in Figure 4a). These distances were evaluated across a range of threshold values from 0.3 to 1.0 nm (Section S3III).

All analyses were performed on equilibrated MD trajectories. Standard GROMACS tools were used to calculate radial distribution functions (RDFs), root-mean-square deviations (RMSDs), *R*_g_, pairwise interaction energies, and end-to-end distances of the PEDOT chains. The conductivity-related parameters derived for PEDOT:PSS systems with various chemical components were compared to corresponding values from reference systems: PEDOT:PSS wet film or dry film, as appropriate. Average values of conductivity-related properties were calculated from data extracted at the end of each half annealing cycle during the final five annealing cycles of each system. For other properties (e.g., end-to-end, *R*_g_, etc.), averages were obtained from NPT simulation data, as described in the corresponding sections. The reference data for wet and dry films were taken from our previous studies.^16,19^ Final equilibrated structures were also visually examined using VMD.^83^

The parameters for dopamine and PEG-400, along with the equilibrated structures of PEDOT-rich and PSS-rich phases containing electrolytes, dopamine and PEG-400, are available in a public repository.^84^ Force-field parameters can be found in a separate repository.^85^

## 3 Results and Discussion

This study investigates how four key conductivity-related morphological parameters, outlined at the end of the previous section, are influenced by three types of chemical components (i.e., aqueous salts/electrolytes, a small biomolecule, and a polar solvent) and how the resulting structural changes correlate with experimental conductivity trends, providing molecular-level insights into the underlying conductivity mechanisms.

Before predictions about the influence of chemical components on conductivity-related parameters are made, it is essential to ensure that the systems have reached equilibration. To assess this, the RMSD of the PEDOT and PSS chains relative to their initial configurations (Figure S4 in the SI), and two key morphological properties: the total number of PEDOT lamella crystallites and their average size (Figures S5 and S6) were monitored over the imulation time. The attainment of a plateau in RMSD values indicates that the systems achieved adequate equilibration (Figure S4 in the SI). In both the PEDOT-rich and PSS-rich phases with chemical constituents, RMSD values stabilized around 5–8 nm, about twice the radius of gyration of PEDOT (∼1.3 nm) and PSS (∼1.8 nm) under dry-film conditions.^16^ This suggests that the polymer chains exhibited sufficient mobility to explore the conformational space and reach equilibrium. Over the same time frame (shaded regions in Figures S5 and S6), morphological parameters also fluctuated around stable values, providing further evidence of equilibration across all systems.

### 3.1 Analysis of Morphological Parameters

Using the last 5 relaxation cycles, we calculated the average lamella crystallite size, normalized count of π–π stacked pairs, nematic order parameter, and normalized interlamella contacts in each phase for three types of chemical constituents. These morphological parameters are closely linked to conductivity: large lamella crystallites facilitate more efficient charge transport in PEDOT:PSS,^86^ increased intra- and interchain π–π stacking enhance charge transport,^87,88^ and improved connectivity between PEDOT chains or PEDOT-rich regions contributes to conductivity enhancements in PEDOT:PSS.^21,89^ To compare the influence of chemical components, data from PEDOT:PSS systems without any chemical entities and those containing water alone are shown in Figure 3. Systems containing dopamine or electrolytes were compared to the hydrated phases (i.e., phases containing only water: wet PEDOT-rich and wet PSS-rich) (Figure 3a–c), while systems containing PEG-400 were compared to PEG-400 free phases (i.e., dry PEDOT-rich and PSS-rich phases) (Figure 3d–f), providing a consistent and rational basis for comparison. Overall, Figure 3 provides a broader overview of the morphological changes induced by the three classes of chemical constituents. In the following sections, we examine each case in more detail, referring back to this figure as needed. For clarity, the normalization procedure for π–π stacked pairs and interlamella contacts was carried out as follows: the number of π–π stacked pairs was divided by the total number of PEDOT chains in the system, while the total number of interlamella contacts was divided by four (corresponding to the four sp^2^ carbon atoms per EDOT unit) and by the number of lamella crystallites, including the individual chains present in each system.

**Figure 3:**
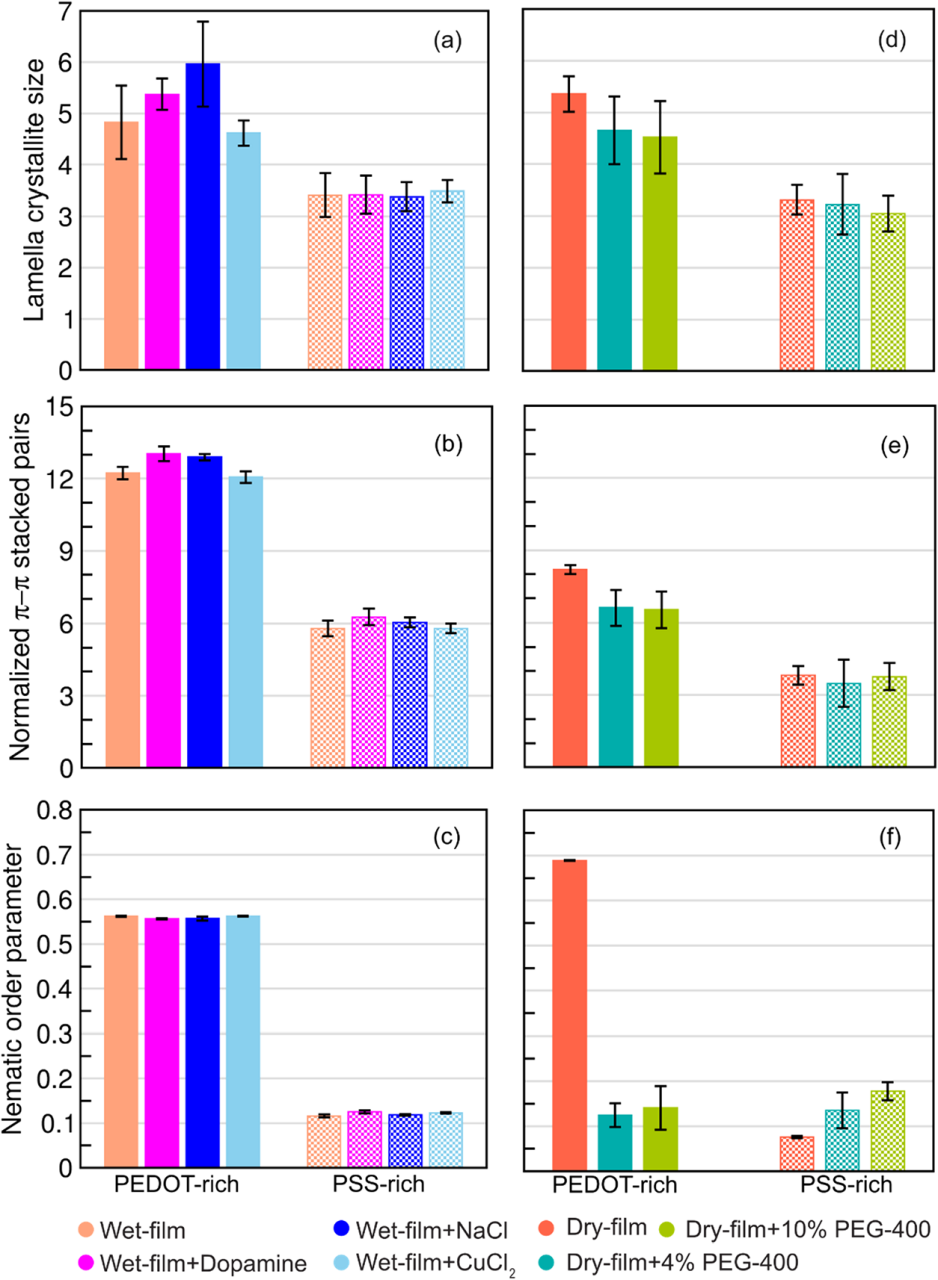
Morphological parameters in the presence of dopamine, NaCl, and CuCl_2_ (a–c) and PEG-400 (d–f). Data for wet and dry films with PEDOT-rich and PSS-rich phases are also shown for comparison. Error bars represent the standard deviation calculated over the last five cycles of data from the current study.

#### 3.1.1 PEDOT-Rich and PSS-Rich Phases with Electrolytes

We first investigated the influence of NaCl and CuCl_2_ on morphological properties relevant to conductivity to evaluate the effect of electrolytes on the PEDOT:PSS conductivity. Three morphological parameters associated with conductivity are presented in Figure 3a–c, alongside corresponding data for the water-diffused PEDOT-rich and PSS-rich phases. In PEDOT lamella crystallites, sp^2^ carbon atoms from one lamella can come in close proximity to those from neighboring lamellae (see the image shown in Figure 4a) without forming π–π stacking interactions. To quantify these potential charge-transfer sites, distances between sp^2^ carbon atoms across adjacent lamellae were computed by analyzing all lamellae and individual chains over a range of distance thresholds (d_t_) from 0.3 to 1.0 nm. The resulting normalized interlamellar contact data for PEDOT-rich and PSS-rich phases in the presence of NaCl and CuCl_2_ are shown in Figure 4a,b, respectively. Higher values for interlamella contacts at a given *d*_t_ indicate improved interlamella connectivity between adjacent crystallites.

**Figure 4:**
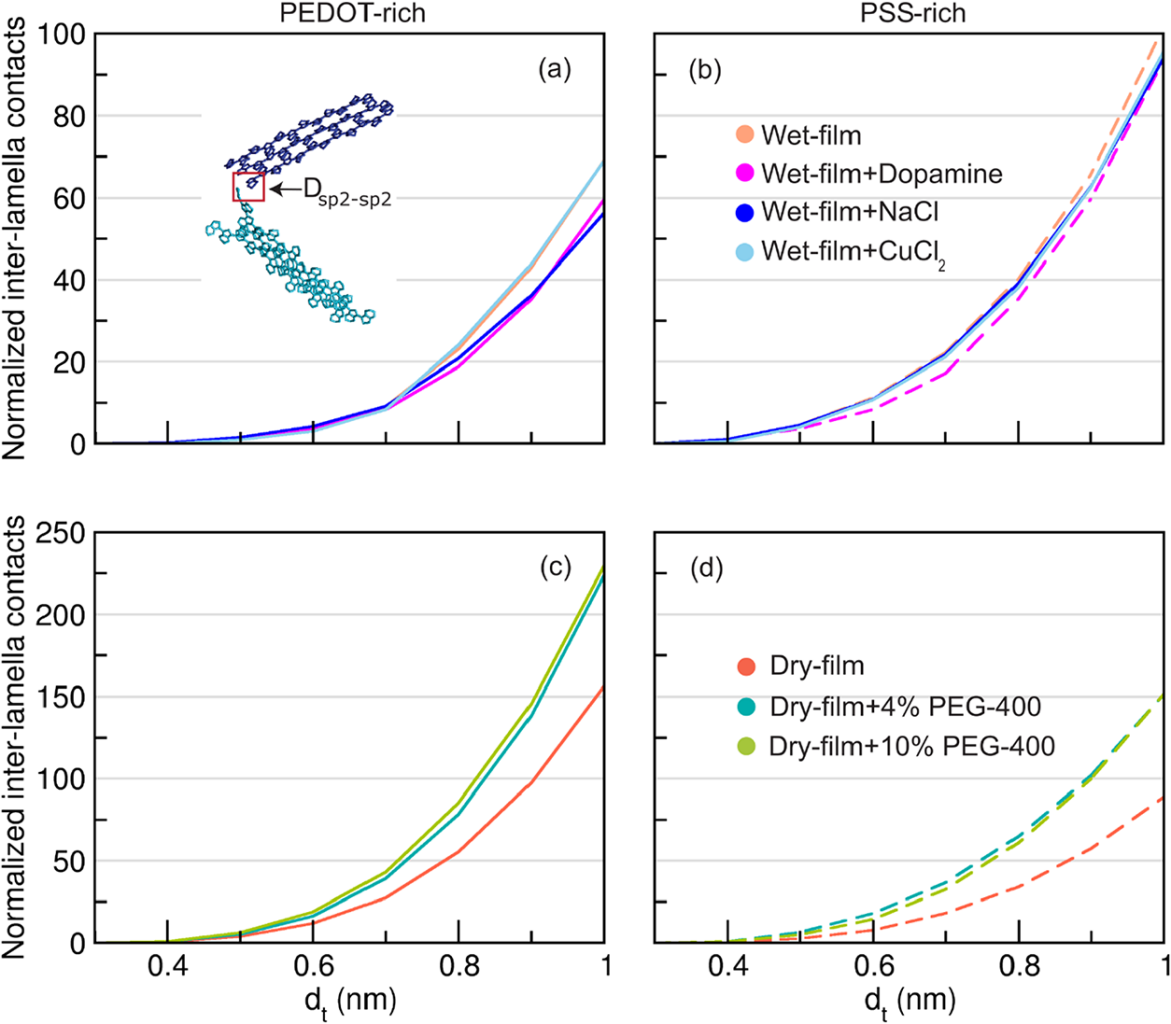
Number of interlamella contacts from non-π–π stacking as a function of *d*_t_ in the presence of dopamine, NaCl, and CuCl_2_ in (a) PEDOT-rich and (b) PSS-rich phases. An example of a non-π–π stacking contact between two PEDOT lamellae (blue and cyan) is shown in panel (a), where *D*_sp2–sp2_ indicates the distance between a sp^2^ carbon atom in the blue lamella and a sp^2^ carbon atom in the cyan lamella.^19^ Interlamella contacts count from non-π–π stacking as a function of *d*_t_ in the presence of PEG-400 in (c) PEDOT-rich and (d) PSS-rich phases.

For NaCl in the PSS-rich phase, no significant changes were observed in any of the morphological parameters analyzed in Figures 3 and 4. In contrast, the PEDOT-rich phase with NaCl showed a slight increase in both the lamella crystallite size and the number of π–π stacked pairs compared to the corresponding wet film. This suggests that the presence of NaCl leads to slightly larger PEDOT crystallites, which in turn enhances intracrystalline π–π stacking, as reflected in the increased number of π–π stacked pairs shown in Figure 3b. However, the number of interlamella contacts arising from non-π–π-stacked interactions was noticeably reduced in the PEDOT-rich phase (Figure 4a), indicating that NaCl diffusion may cause the lamellae to move farther apart. This separation reduces interlamella connectivity, which plays a critical role in the overall charge transport ability of the material.^90^ Thus, although the slight increase in lamella crystallite size results in improved π–π stacking, which may enhance charge transfer within crystallites, this benefit appears minimal and offset by the reduction in interlamella contacts. Experimentally, NaCl treatment is known to have little or no effect on enhancing the conductivity.^36^ Since our simulations showed a noticeable decrease in interlamella contacts alongside only a marginal increase in crystallite size, we argue that no meaningful morphological change occurs that could account for conductivity enhancement, in agreement with experimental findings.

For CuCl_2_, no significant changes were observed in any of the morphological parameters shown in Figure 3 for either the PEDOT-rich or PSS-rich phases. However, unlike NaCl, CuCl_2_ did not reduce the number of interlamella contacts in the PEDOT-rich phase, instead it maintained values comparable to those of wet film (Figure 4a). This suggests that no morphological parameter directly explains the conductivity modulation of the material. Experimentally, however, CuCl_2_ treatment is known to enhance PEDOT:PSS conductivity, a mechanism generally attributed to charge screening, which weakens the Coulombic attraction between PEDOT and PSS, leading to partial PSS removal and PEDOT conformational changes,^36^ factors that collectively contribute to improved conductivity. To further probe the screening effect, we computed RDFs between Cu^2+^ and an O_2_ atom of the sulfonate groups in PSS (as defined in the itp file^84^) and between Na^+^ and the same oxygen atom, in both PEDOT-rich and PSS-rich phases (Figure S7a). In both phases, Cu^2+^ and Na^+^ exhibit pronounced first-shell peaks at approximately 0.18 and 0.22 nm, respectively, with Cu^2+^ showing significantly higher peak intensities. This indicates stronger interactions between Cu^2+^ and the sulfonate oxygen atoms of PSS compared to those between Na^+^ and PSS. These observations are consistent with the notion that metal ions with a positive softness parameter (e.g., Cu^2+^) strongly bind to PSS, as reported in the literature, supporting the view that CuCl_2_-induced conductivity enhancement arises primarily from the strong screening effects of Cu^2+^.^36^ However, since our MD data showed no significant changes in conductivity-related morphological parameters, this suggests that charge screening alone cannot fully explain the conductivity enhancement. Instead, secondary morphological changes such as PEDOT conformational changes associated with partial PSS loss, as described in previous studies,^36^ are likely required to explain the observed conductivity improvement. Incorporating PSS loss into the material model may therefore be the key to uncovering the underlying mechanism.

#### 3.1.2 PEDOT-Rich and PSS-Rich Phases with Dopamine

Next, we investigated the influence of dopamine diffusion into the PEDOT-rich and PSS-rich phases and their impact on conductivity-related properties (Figures 3 and 4). Among the four properties examined, the lamella crystallite size, number of π–π stacked pairs, orientation parameter, and number of interlamella contacts, only the latter showed a noticeable effect. Specifically, we observed a significant reduction in the number of interlamella contacts in the PEDOT-rich phase and a marginal reduction in the PSS-rich phase compared to the corresponding water-diffused systems. This suggests that dopamine diffusion into the material could possibly decrease the conductivity. To the best of our knowledge, structure-conductivity data for aqueous dopamine diffusion into PEDOT:PSS is lacking in the literature; however, doping with dopamine hydrochloride, which contains protonated dopamine, has been reported to enhance PEDOT:PSS conductivity by improving molecular packing.^91^ Even though dopamine in our simulation was in a neutral form and cannot form electrostatic interactions with PSS to improve the molecular packing, we investigated the possibility of forming hydrogen bonds with PSS. To explore this, we calculated RDFs between the hydroxyl hydrogens of dopamine (H4 and H5 in the itp file^84^) and one of the oxygen atoms of the sulfonate group in PSS (i.e., O_2_), within PEDOT-rich and PSS-rich phases, as shown in Figure S7b in the SI. The sharp peaks at ∼0.17 nm confirm the presence of hydrogen bonding between atoms considered. Visual inspection of the PEDOT-rich phase with dopamine (Figure 5b) reveals that dopamine intercalates between PEDOT and PSS. Thus, our MD data suggest that dopamine forms hydrogen bonds with PSS and disrupts interdomain connectivity. We propose that this disruption is responsible for the reduced interlamella contacts observed in our MD simulations. Therefore, while the charged form of dopamine may enhance PEDOT:PSS conductivity, we argue that dopamine diffusion through an aqueous medium could instead reduce conductivity by impairing interlamella connectivity.

**Figure 5:**
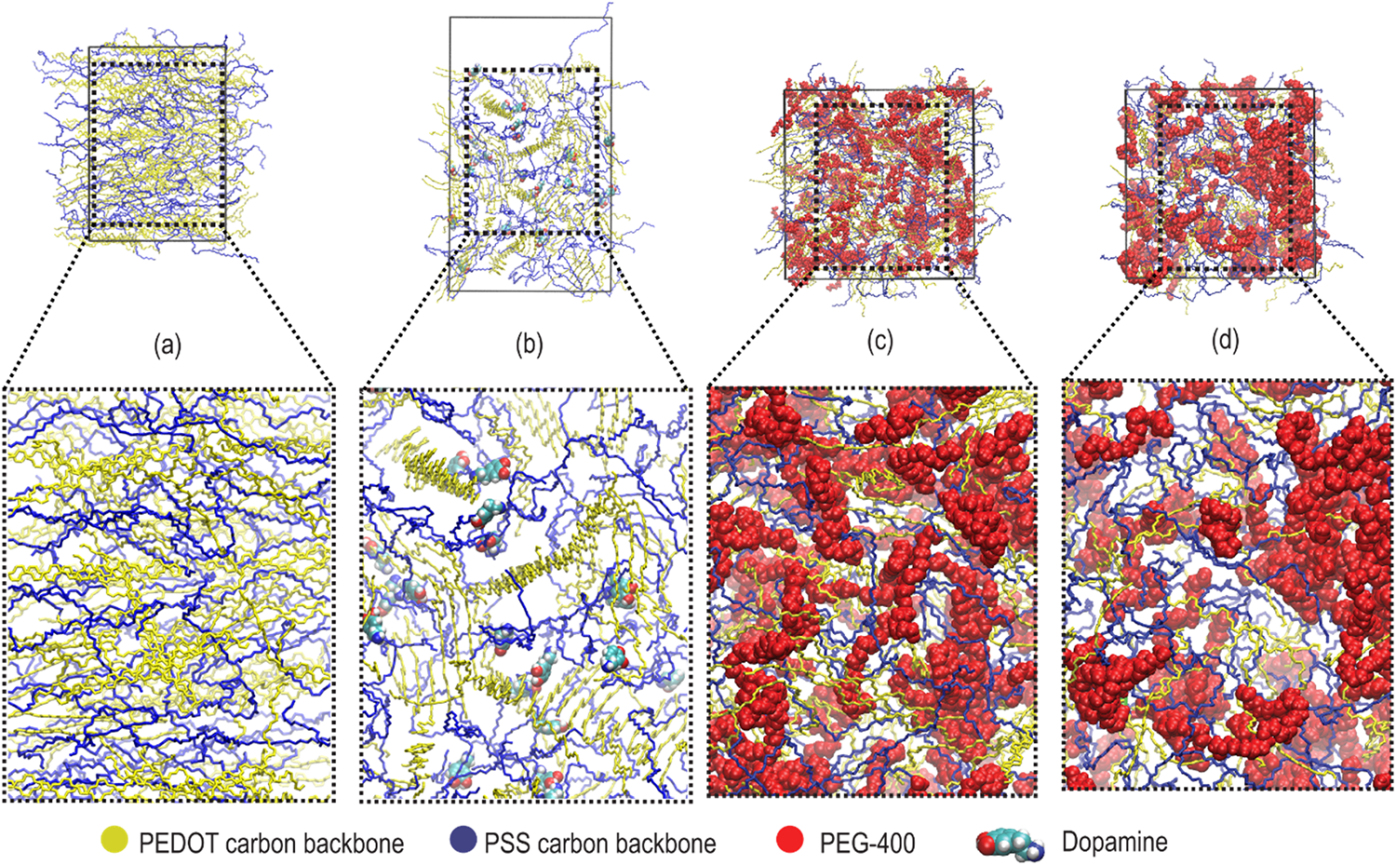
Final frames of simulations: (a) PEDOT-rich phase without any additional chemical components (dry PEDOT-rich phase), (b) dopamine intercalated between PEDOT and PSS in the PEDOT-rich phase, (c) PEG-400 intercalated in the PEDOT-rich phase with 10% v/v PEG-400, and (d) PEG-400 intercalated in the PSS-rich phase with 10% v/v PEG-400. Water molecules are omitted for the sake of clarity.

#### 3.1.3 PEDOT-Rich and PSS-Rich Phases with PEG-400

Lastly, we investigated how the presence of PEG-400 influences conductivity-related properties in both PEDOT-rich and PSS-rich phases. Our results (Figure 3d–f) show that PEG-400 induces a slight reduction in the average PEDOT crystallite size and the number of π–π stacked pairs within the PEDOT-rich phase, with no further changes observed upon increasing the PEG-400 concentration, compared to the PEG-free system. However, these reductions are not significant, and no such trend was observed in the PSS-rich phase. In contrast, the nematic order parameter in the PEDOT-rich phase decreases significantly with PEG-400 (Figure 3f), indicating that the highly ordered PEDOT arrangement in the PEG-free system was disrupted with no notable difference between the two PEG-400 concentrations. The alignment of PEDOT chains in the PEDOT-rich phase with 4% v/v PEG-400, as well as in the corresponding phase containing NaCl and dopamine (Figure S8), visually corroborates the trends observed in Figure 3c,f for the PEDOT-rich phase. Conversely, in the PSS-rich phase, PEG-400 slightly increases the nematic order parameter, indicating that the isotropic dry PSS-rich phase adopts partially ordered orientations in its presence. Although the increase in PEG-400 concentration is minor and not significant, it reflects a modest alignment of PEDOT chains that could facilitate enhanced local charge transport compared to that of the dry system. Nevertheless, the overall nematic order parameter remains low, indicating that PEDOT chains are not strongly aligned in the presence of PEG-400, consistent with the trend in the PEDOT-rich phase. Meanwhile, interlamella contacts increased significantly in both PEDOT-rich and PSS-rich phases with PEG-400 (Figure 4c,d), suggesting closer crystallite connectivity. Notably, although the dry PSS-rich phase is inherently less conductive due to its more isotropic and poorly connected PEDOT network compared to the dry PEDOT-rich phase,^16,19^ the introduction of PEG-400 promotes a more interconnected PEDOT network in the PSS-rich phase. This enhancement appears independent of PEG-400 concentration, as both 4% (v/v) and 10% (v/v) PEG-400 show similar levels of contact in both phases. Although the slight reduction in crystallite size and π–π stacking, along with the significant drop in the nematic order parameter (i.e., PEDOT-rich phase), could suggest reduced conductivity, this is not the case, as PEG-400 results in a marked improvement in conductivity.^55^

Literature reports attribute the conductivity enhancement from PEG treatment to its ability to screen electrostatic interaction between PEDOT and PSS through hydrogen bonding with PSS. This promotes phase separation and induces a conformational change of PEDOT chains from coiled to more linear structures (Scheme 1 in ref 55), thereby facilitating interdomain charge hopping.^55^ To assess the reorientation of PEDOT chains, we compared the PEDOT-rich phase in the absence and presence of 10% PEG-400 by evaluating the radius of gyration and the end-to-end distance of PEDOT chains using the final 50 ns of the NPT trajectories. In the PEG-free PEDOT-rich phase, the average *R*_g_ and end-to-end distance were 1.3081 ± 0.0006 nm and 4.3450 ± 0.0033 nm, respectively. For the PEDOT-rich phase containing 10% v/v PEG-400, these values were 1.2970 ± 0.0006 and 4.2638 ± 0.0036 nm, respectively. The minimal differences between the two systems indicate that coil-to-linear reordering of PEDOT chains is not substantially evident. Similar behavior is observed in the PSS-rich phase at two PEG concentrations, as summarized in Table S5 of the Supporting Information. We next examined hydrogen bond formation induced by PEG-400 and the consequent screening effects it may produce. We calculated RDFs between the terminal polar hydrogen atoms (H1 and H38 in the itp file^84^) of PEG-400 and one of the oxygen atoms of the sulfonate groups in PSS (i.e., O2, as defined in the itp file^84^) for both PEDOT-rich and PSS-rich phases at the two PEG-400 concentrations, as shown in Figure S7c,d in the SI. The sharp peaks observed around 0.17 nm in all four systems indicate the formation of hydrogen bonds between PEG-400 and PSS. This interaction weakens the electrostatic attraction between PEDOT and PSS, consistent with both the screening effect and hydrogen bonding reported in the literature.^55^

To further investigate the changes in the spatial distribution of PEDOT and PPS induced by PEG-400, we calculated the RDFs between sulfur atoms along PEDOT chains (Figure S9a) and between sulfur atoms in PSS sulfonate groups and PEDOT chains (Figure S9b) as shown in the Supporting Information. Similar calculations were also performed for other chemical components studied. Figure S9a shows that PEG-400 reduces interactions between PEDOT chains, leading to weaker local ordering compared with other chemical components studied. This effect is more pronounced in the PEDOT-rich phase than in the PSS-rich phase (systems with 10% v/v PEG-400), as evidenced by the reduced intensity of the first two RDF peaks. Figure S9b exhibits a similar trend, indicating diminished PEDOT–PSS interactions upon the addition of PEG-400. However, this effect is weak and not clearly distinguishable from the RDF alone. To directly assess the impact of PEG-400 on PEDOT–PSS interactions, we calculated pairwise electrostatic and van der Waals interaction energies for PEDOT and PSS in both PEDOT-rich and PSS-rich phases at 300 K, comparing the 10% v/v PEG-400 system with the corresponding PEG-free systems (Figure S10). Incorporation of PEG-400 reduces the magnitude of the electrostatic interaction energies in both phases (Figure S10a,b), reflecting a reduction in attractive forces between PEDOT and PSS. A similar trend is observed for van der Waals interaction energies (Figure S10c,d), confirming that PEG-400 weakens both electrostatic and van der Waals interactions between the polymer chains. Visual inspection of the final simulation frames for the PEDOT-rich and PSS-rich phases with 10% (v/v) PEG-400 show that PEG-400 molecules intercalate between PEDOT and PSS (Figure 5c,d), promoting separation of the two components and corroborating the results discussed above. Comparison of PEDOT and PSS arrangements in the absence (Figure 5a) and presence of PEG-400 (Figure 5c,d) further illustrates PEG-400’s role in driving this separation.

As our MD data suggest that PEG-400 enhances intercrystallite connectivity without significantly altering the overall chain conformation, we propose that by reducing the electrostatic and van der Waals interactions between PEDOT and PSS, PEG-400 may allow PEDOT crystallites to move closer together or adopt orientations that promote closer contacts between neighboring lamella crystallites. Interestingly, this connectivity enhancement is observed even in the less conductive PSS-rich phases in the presence of PEG-400. This mechanism could still contribute to improved charge transport by facilitating interdomain hopping pathways, representing an alternative or complementary explanation to the widely discussed coil-to-linear reordering model.^55^

## 4 Conclusions

Using atomistic models of PEDOT-rich and PSS-rich phases, we performed molecular dynamics simulations to investigate how different chemical constituents: electrolytes, dopamine, and PEG-400, affect morphological parameters related to conductivity. Our MD simulations reveal that morphological changes are sensitive to both the chemical constituent and the phase arrangement. We observe that interlamellar connectivity is particularly susceptible to modulation and plays a significant role in conductivity changes, whereas factors such as PEDOT lamella crystallite size and chain orientation are less affected by the chemical constituents studied (except PEG-400). Among the chemical components studied, dopamine intercalates between PEDOT and PSS, disrupting interdomain connectivity and potentially reducing conductivity due to the diffusion of aqueous dopamine into PEDOT:PSS. In contrast, PEG-400 enhances conductivity by improving interlamellar connectivity without altering PEDOT chain conformation. Notably, this enhancement is observed even in the less conductive PSS-rich phase, although it is more pronounced in the PEDOT-rich regions. No other conductivity-related morphological parameters show a measurable contribution to conductivity enhancement in PEG-400-containing systems. CuCl_2_ increases conductivity via PEDOT conformational changes associated with partial PSS loss, whereas electrostatic screening alone produces minimal morphological changes affecting the conductivity. Overall, our MD results support established findings such as NaCl having a minimal effect on conductivity enhancement and CuCl_2_ enhancing conductivity through PEDOT conformational changes and PSS loss. They also offer alternative interpretations, such as PEG-400 improving interlamella connectivity without altering PEDOT chain orientation, challenging the conventional view that conductivity enhancement arises from coil-to-linear ordering of PEDOT chains. These observations highlight the value of MD simulations as a powerful tool for validating or re-evaluating existing mechanistic explanations in complex polymer systems.

## Supplementary Material



## Data Availability

The authors declare that all data supporting the findings of this study are available within the paper, ESI, and a public repository.^84^

## Notes

PEG-400 charge-modification methods; validation of PEG-400, dopamine, and ion parameters; annealing protocols used in the study; implementation of analysis tools; RMSD of the PEDOT and PSS chains relative to their initial configurations as a function of simulation time for each chemical constituent; total number of PEDOT lamella crystallites as a function of simulation time for each chemical constituent; average number of PEDOT lamella crystallite size as a function of simulation time for each chemical constituent; RDFs calculated between relevant atoms for systems in PEDOT-rich and PSS-rich phases under each chemical constituent; PEDOT chain arrangement in the PEDOT-rich phase containing NaCl, dopamine, and 10% PEG-400; radius of gyration and end-to-end distance data for PEDOT chains in PEG-free systems and in PEG-containing PEDOT-rich and PSS-rich phases; RDFs between sulfur atoms within PEDOT chains and between PEDOT and PSS; Coulombic and Lennard-Jones short-range interaction energies between PEDOT and PSS in both PEDOT-rich and PSS-rich phases, compared with their corresponding PEG-free systems and with systems containing 10% PEG-400 (PDF)
