# Supplementary material for: Morphological Changes in PEDOT:PSS under Electrolytes, Dopamine, and PEG-400 Exposure: A Molecular Simulation Perspective
Source: Macromolecules. 2026 Jan 27;59(3):1752–62. doi: 10.1021/acs.macromol.5c02727 (PMC12895523; doi:10.1021/acs.macromol.5c02727)
Supplement: Supplementary file 1 [file ma5c02727_si_001.pdf]

Morphological Changes in PEDOT:PSS under Electrolytes, Dopamine, and PEG-400 Exposure: A Molecular Simulation Perspective

Amali G. Guruge[a]*, Hesam Makki[a] and Alessandro Troisi[a]

[a]Department of Chemistry, University of Liverpool, Liverpool L69 3BX, UK.

Email: amali.galappaththi-guruge@liverpool.ac.uk

Section S1 – Atomic Charges for PEG-400

Classical force fields generally assign consistent atomic charges to chemically equivalent atoms in polyethylene glycol (PEG) systems.[1, 2] However, when using PolyParGen derived parameters[3], we observed inconsistencies in the charges assigned to ethylene glycol repeat units (Table S1). To evaluate how these charge differences influence physical properties, we compared two PEG-400 models: one using the original PolyParGen-derived charges (i.e., unchanged), and another in which the charges were adjusted to enforce consistency among chemically equivalent atoms (i.e., modified charge model in Table S1).

To generate consistent charges, we first optimized a short PEG oligomer using Gaussian16[4] at the B3LYP/6-31G* level of theory, and computed atomic charges using the CHELPG scheme.[5] These charges were then transferred to PEG-400. Chemically equivalent atoms (e.g., ether oxygens, terminal hydroxyl oxygen, etc.) were identified, and their charges were averaged and reassigned to the corresponding atoms, resulting in a PEG-400 model with consistent atomic charges (Table S1). The following section describes the calculation of the physical properties of PEG-400 using both the original (PolyParGen-assigned) and modified charge models. It is important to note that all other parameters (e.g., bond lengths, angles, and dihedrals) from the PolyParGen-assigned model were retained unchanged in the modified charge model.

Table S1. Charges obtained from PolyParGen and from the modified charge model used in this study.

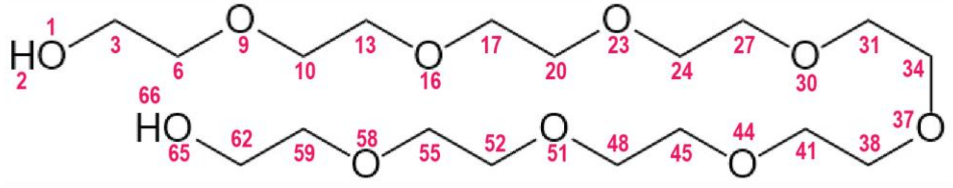

Atom number	Atom	PolyParGen	Modified charged model
1	O	-0.39399	-0.6557347
2	H	0.210161	0.3929955
3	C	0.066495	0.2501260
4	H	0.058821	0.0118408
5	H	0.058821	0.0118408
6	C	0.06983	0.2501260
7	H	0.059106	0.0118408
8	H	0.059106	0.0118408
9	O	-0.37669	-0.5503823
10	C	0.070106	0.2501260
11	H	0.059116	0.0118408
12	H	0.059116	0.0118408
13	C	0.070107	0.2501260
14	H	0.059116	0.0118408
15	H	0.059116	0.0118408
16	O	-0.37668	-0.5503823

17	C	0.070107	0.2501260
18	H	0.059116	0.0118408
19	H	0.059116	0.0118408
20	C	0.070107	0.2501260
21	H	0.059116	0.0118408
22	H	0.059116	0.0118408
23	O	-0.37668	-0.5503823
24	C	0.070107	0.2501260
25	H	0.059116	0.0118408
26	H	0.059116	0.0118408
27	C	0.070107	0.2501260
28	H	0.059116	0.0118408
29	H	0.059116	0.0118408
30	O	-0.37668	-0.5503823
31	C	0.070107	0.2501260
32	H	0.059116	0.0118408
33	H	0.059116	0.0118408
34	C	0.070107	0.2501260
35	H	0.059116	0.0118408
36	H	0.059116	0.0118408
37	O	-0.37668	-0.5503823
38	C	0.070107	0.2501260
39	H	0.059116	0.0118408

40	H	0.059116	0.0118408
41	C	0.070107	0.2501260
42	H	0.059116	0.0118408
43	H	0.059116	0.0118408
44	O	-0.37668	-0.5503823
45	C	0.070107	0.2501260
46	H	0.059116	0.0118408
47	H	0.059116	0.0118408
48	C	0.070107	0.2501260
49	H	0.059116	0.0118408
50	H	0.059116	0.0118408
51	O	-0.37668	-0.5503823
52	C	0.070107	0.2501260
53	H	0.059116	0.0118408
54	H	0.059116	0.0118408
55	C	0.070106	0.2501260
56	H	0.059116	0.0118408
57	H	0.059116	0.0118408
58	O	-0.37669	-0.5503823
59	C	0.06983	0.2501260
60	H	0.059106	0.0118408
61	H	0.059106	0.0118408
62	C	0.066495	0.2501260

63	H	0.058821	0.0118408
64	H	0.058821	0.0118408
65	O	-0.39399	-0.6557347
66	H	0.210161	0.3929955

Note: Hydrogen atoms bonded to carbon are not shown in the table figure of PEG-400. For example, atom number 4 and 5 correspond to the hydrogen atoms attached to the carbon atom with atomic number 3, and this pattern continues along the entire PEG-400 chain.

Section S2 - Validation of Simulation Parameters Based on Experimental Data and Annealing Protocols

(I). Validation of parameters for PEG-400

Recognizing that modified charges derived from density functional theory calculations (as described in Section 1 of the SI) may not be ideal for PEG-400 in our systems, we further validated them by calculating the density, radius of gyration, and end-to-end distance of PEG-400 in simple reference systems as follows.

To determine the density of PEG-400, we constructed a ~5 nm cubic box containing 200 PEG-400 molecules. For calculation of radius of gyration and end-to-end distance, a separate ~7.5 nm cubic box was prepared with one PEG-400 molecule solvated in 14100 water molecules. MD simulations were run for 200 ns at 1 bar and 296 K using the protocol described in the main paper. Data was extracted from the final portion of the trajectory (i.e., 150-200 ns) at 25 ps intervals. The density, radius of gyration, and end-to-end distance were calculated using standard GROMACS tools. Figures S1a to S1c show the time evolution of these properties over the final 50 ns of the simulations. The corresponding averaged values are reported in Table S2, along with experimental data or previously reported simulation values for comparison. As shown in Table S2, the properties calculated using the modified charges are in good agreement with experimental values. Furthermore, the calculated root mean square error (RMSE) between the two charge models is 0.13, and the correlation coefficient (R^2) of 0.87 indicates that the overall trends in the atomic charges are largely consistent. Therefore, we proceed with the model in which chemically equivalent atoms were assigned similar charges.

Table S2. Comparison of the density, radius of gyration, and end-to-end distance of PEG-400 obtained from molecular dynamics simulations with experimental values or previously reported simulation data.

Physical property	PolyParGen-assigned model	Modified charge model	Experimental/Simulation data
Density (g cm^{-3})	1.163 ± 0.003	1.177 ± 0.003	1.13 (at 293 K)[6]
Radius of gyration (nm)	0.550 ± 0.078	0.559 ± 0.082	0.64 ± 0.01[7]*
End-to-end distance (nm)	1.268 ± 0.449	1.323 ± 0.424	1.55 ± 0.04[7]*

Errors represent the standard deviation calculated from data extracted at 25 ps intervals during the final 50 ns of the simulation. *Data correspond to PEO_9. Note that PEO_1 refers to CH_3-O-CH_2-CH_2-O-CH_3, and PEO_2 is CH_3-O-CH_2-CH_2-O-CH_2-CH_2-O-CH_3.

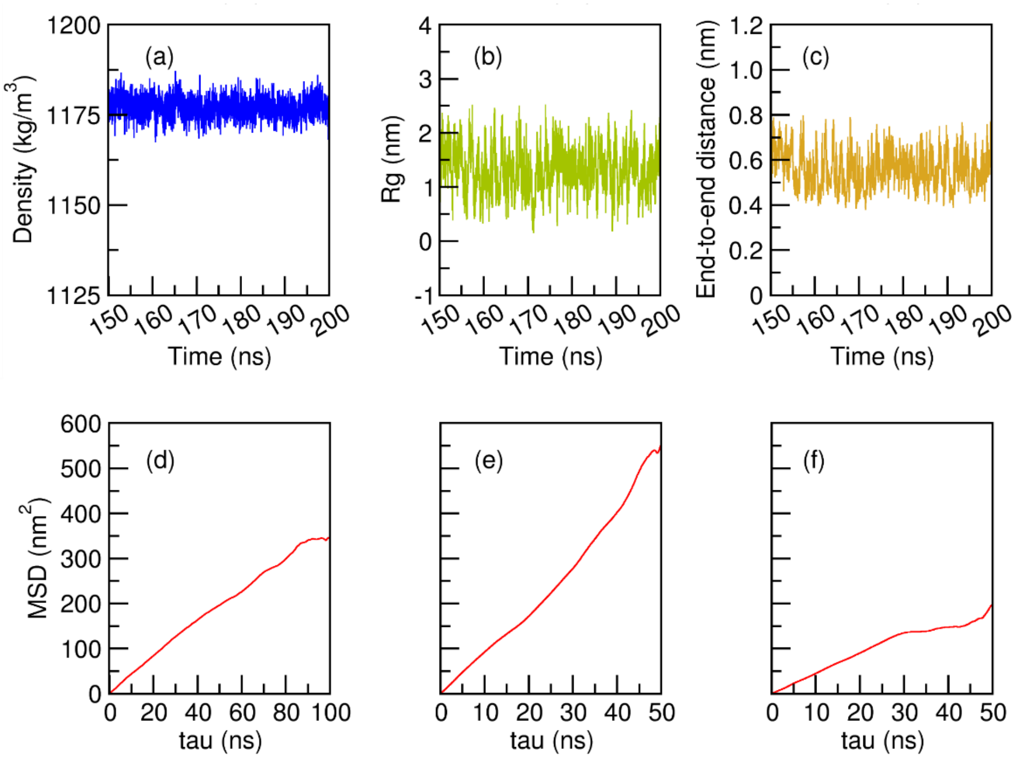

Figure S1. (a) System density of PEG-400 as a function of time, (b) radius of gyration of PEG-400 as a function of time, and (c) end-to-end distance of PEG-400 as a function of time. Mean square displacement (MSD) as a function of time for (d) dopamine, (e) Cl^- ions and (f) Cu^{2+} ions.

(II). Validation of parameters for dopamine and ions

To determine the diffusion coefficient of dopamine in water, we simulated a cubic box containing 10 dopamine and 7220 water molecules, corresponding to a concentration of approximately 0.08 mol dm^{-3}, at 298 K. The simulation was carried out for 200 ns, following the MD protocol described in the Method section of the main paper. Similarly, a system containing 7 Cu^{2+} ions, 14 Cl^- ions, and 7220 water molecules was constructed in a cubic box and simulated for 300 ns under the same protocol used for the dopamine-water system. In both cases, the diffusion coefficients of dopamine, Cu^{2+} and Cl^- were calculated using the *gmx_msd* tool in GROMACS.[8] The diffusion coefficients were obtained by fitting the linear region of the mean square displacement (MSD)

curves (Figure S1d to S1f) for each molecular component. The calculated values were then compared with available experimental data, as summarized in Table S3.

Table S3. Comparison of diffusion coefficients calculated from molecular dynamics simulations with experimental values.

Molecule/Ion	Diffusion coefficient ($\times 10^{-5}$ cm^2 s^{-1})	
	From MD	Experimental value
Dopamine	0.6911 ± 0.0355	0.605 ± 0.025[9] *
Cu^{2+}	0.7658 ± 0.0350	0.714[10]
Cl^-	1.9569 ± 0.3159	2.03[10]

* In 0.1 M, pH 7.4 phosphate buffer at 298 K.

(III). Annealing protocols

Table S4. Annealing protocols used the study.

Moderate-temperature annealing protocol							
Simulation time (ns)	0	1	3	13	23	24	25
Annealing temperature (K)	310	325	360	360	360	325	310
High-temperature annealing protocol							
Simulation time (ns)	0	1	11	13	23	25	26
Annealing temperature (K)	300	1100	1100	800	800	300	300

Section S3 - Implementation of analysis tools

The section describes the morphological analysis performed in the study. Unless otherwise noted, all analyses were carried out using the final five frames obtained from the annealing cycles of each system.

I. Lamella crystallite size, and number of π-π stacked pairs – We used *pistacking.py* script (*https://github.com/HMakkiMD/PEDOT-PSS/blob/main/Pistacking-git/pistacking.py*) from a previous study.[11] This script defines the plane formed by the heavy atoms of each EDOT monomer and identifies π-π stacked EDOT pairs on different chains based on three criteria[11]; i) the angle between the normal vectors of the two EDOT planes is less than 10°, ii) the π-π stacked distance ($D_{\pi\text{-}\pi}$ shown in Figure S2) is less than 0.4 nm and iii) the horizontal distance between the center of geometry of each parallel pair is less than 0.5 nm. Additionally, the script accounts for PEDOT chains that share at least one π-π stacked EDOT pair belonging to a single PEDOT lamella cluster. This allows for quantification of number of PEDOT chains per lamella cluster (i.e., lamella crystallite size) and the total number of lamella crystallites present in each phase.

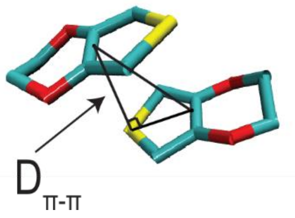

Figure S2. Example of a π-π stacked EDOT pair, where $D_{\pi\text{-}\pi}$ denotes the π-π stacking distance. Carbon atoms are shown in cyan, sulfur in yellow, and oxygen in red.

II. Orientation parameter of PEDOT – We employed a python script to compute the nematic order parameter *S*. The script extracts PEDOT sulfur atom coordinates from a PDB file and constructs an end-to-end orientation vector for each polymer chain by summing consecutive sulfur–sulfur segment vectors. These chain vectors are then normalized in the calculation, and the nematic order parameter along with the corresponding director are obtained from the nematic tensor *Q*, following standard approaches for orientational order in liquid crystalline systems[12], where *S* is the largest eigenvalue of *Q* and the director is its associated eigenvector. For each system, the final five frames were used to calculate *S*, and the mean value along with the standard deviation is reported in Figure 3c and 3f.

Prior to the application, the python code was validated using a noise test to assess the sensitivity of the nematic order calculation. PEDOT chains were initially perfectly aligned along the x-axis of the simulation box, yielding a nematic order parameter of S=1.0000 and a corresponding director of [-0.999, -0.055, -0.000], confirming correct identification of orientational order. Random angular perturbations drawn from a Gaussian distribution with increasing standard deviations (σ up to 90°) were then applied to the chain orientation vectors. This led to a progressive decrease in the nematic order parameter to values near S ≈ 0.25, reflecting substantial loss of orientational order. Figure S3a shows how the nematic order parameter decreases as the standard deviation of the applied Gaussian angular noise increases from 0° to 90°. These tests confirm that the code reliably generates orientation vectors for each chain, accurately captures the degree of alignment, and is sensitive to deviations from perfect order. Representative chain orientation vectors (prior to normalization) for aligned and noisy systems are shown in Figure S3a and S3b. The noisy

system corresponds to the dry PSS-rich phase with dopamine, where S = 0.125 and the director is [0.57062492 0.44943803 -0.68730827]

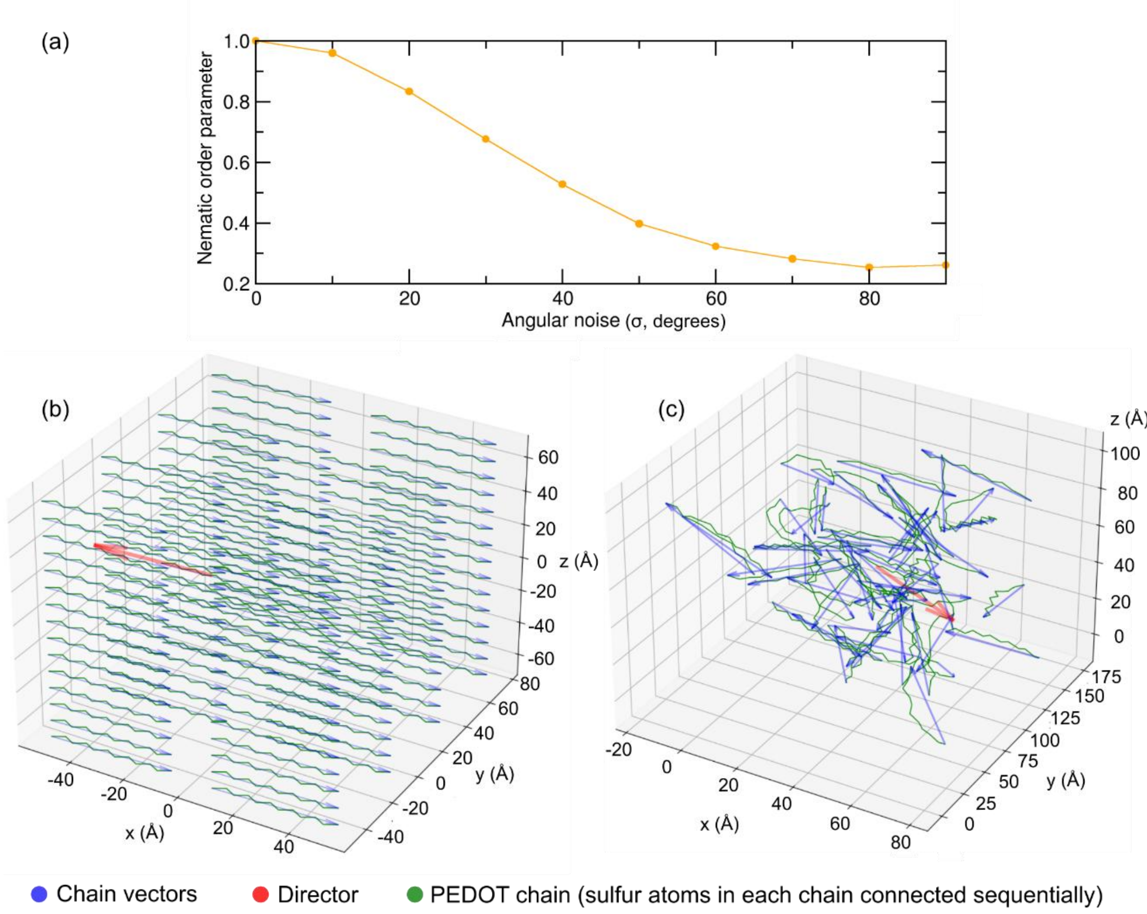

Figure S3. (a) Nematic order S as a function of the standard deviation σ of Gaussian angular noise (σ = 0° to 90°). Chain orientation vectors for (b) an aligned PEDOT chains configuration and (c) a randomly oriented PEDOT chains configuration from the dopamine containing PSS-rich phase. In each case, the corresponding nematic director calculated from the chain orientation vectors is shown in red.

III. Connectivity between lamella crystallites – We employed the *Inter-lamellae.py* script (https://github.com/HMakkiMD/PEDOT-PSS/blob/main/Inter-lamellae-contact-git/Inter-lamellae.py) to analyze the connectivity between lamella crystallites.[11] The script calculates the shortest distance between sp^2 carbon atoms ($D_{sp2\text{-}sp2}$) belonging to two neighboring lamella crystallites (See the illustration in Figure 4a). Connectivity of all lamella clusters and individual PEDOT chains was evaluated by comparing this shortest distance to a threshold value, varied within the typical electron tunneling range of 0.3-1.0 nm.[11]

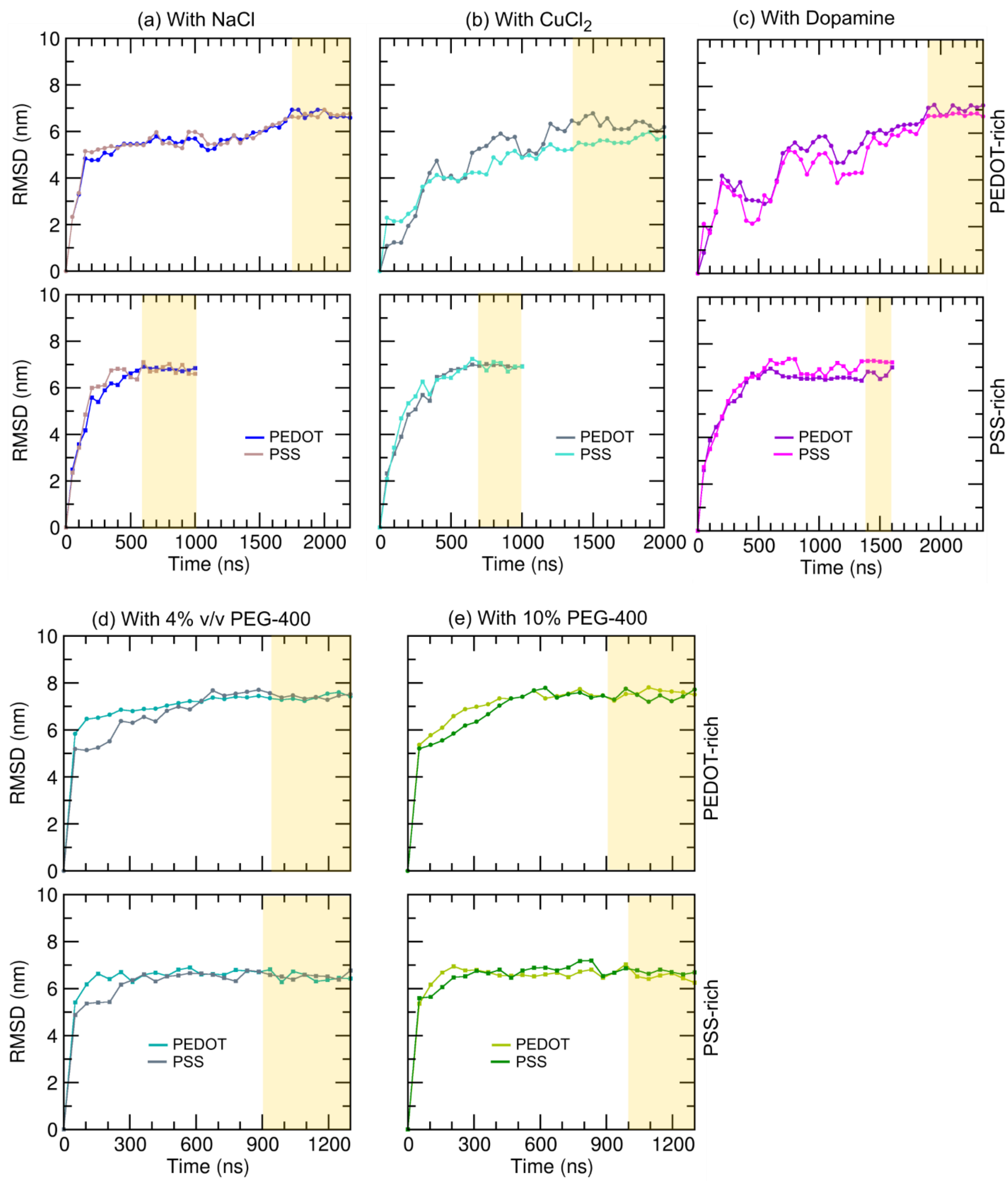

Figure S4. Root-mean-square deviation (RMSD) of the PEDOT and PSS chains relative to their initial configurations as a function of simulation time for each chemical constituent. The shaded regions indicate the equilibrated regime. Under each chemical constituent, the top panels correspond to the PEDOT-rich phases, while the bottom panels show the corresponding PSS-rich phases.

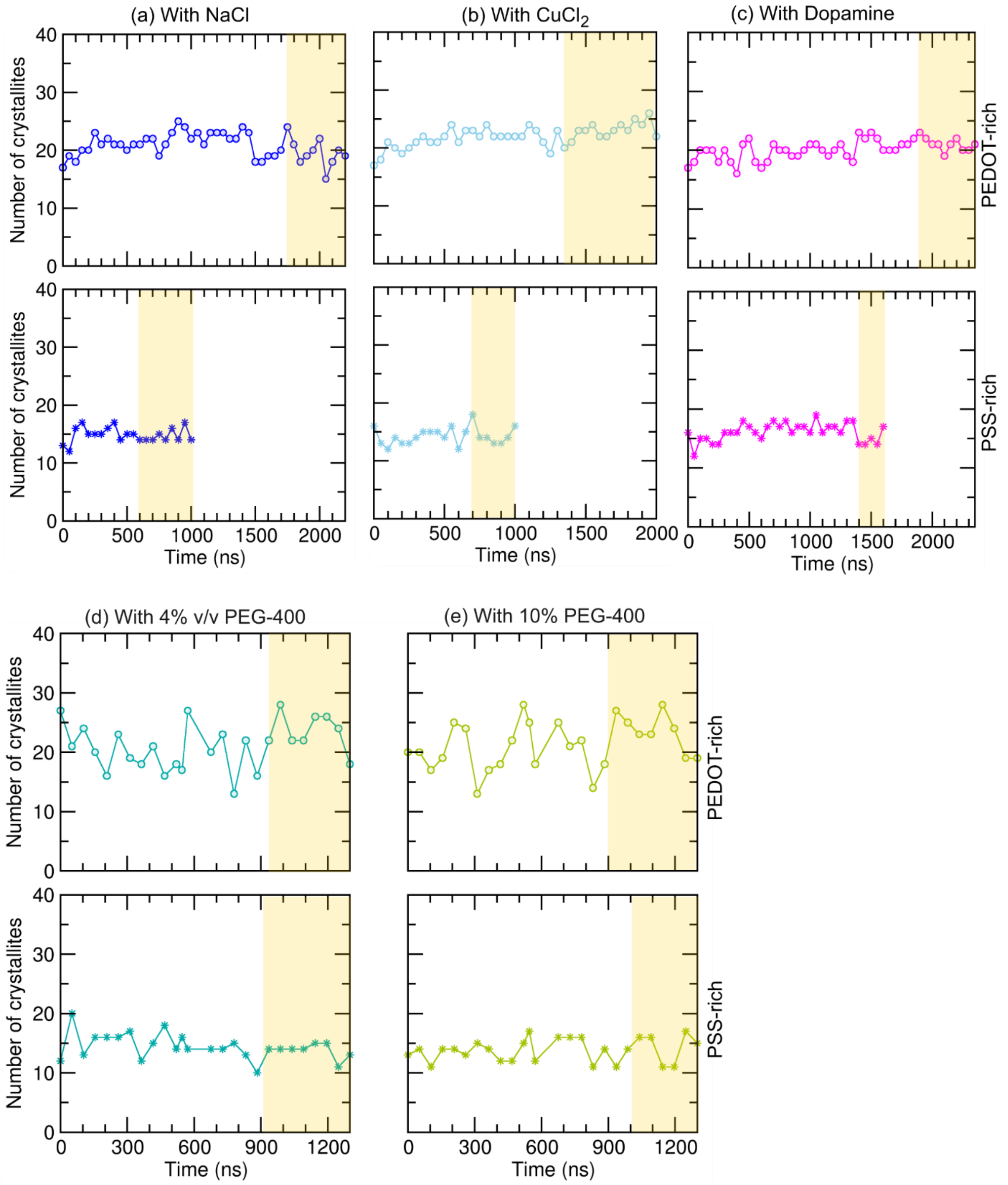

Figure S5. Total number of PEDOT lamella crystallites formed over the simulation time for each chemical constituent. The shaded region indicates the equilibrated regime. Under each chemical constituent, the top panels correspond to the PEDOT-rich phases, while the bottom panels show the corresponding PSS-rich phases.

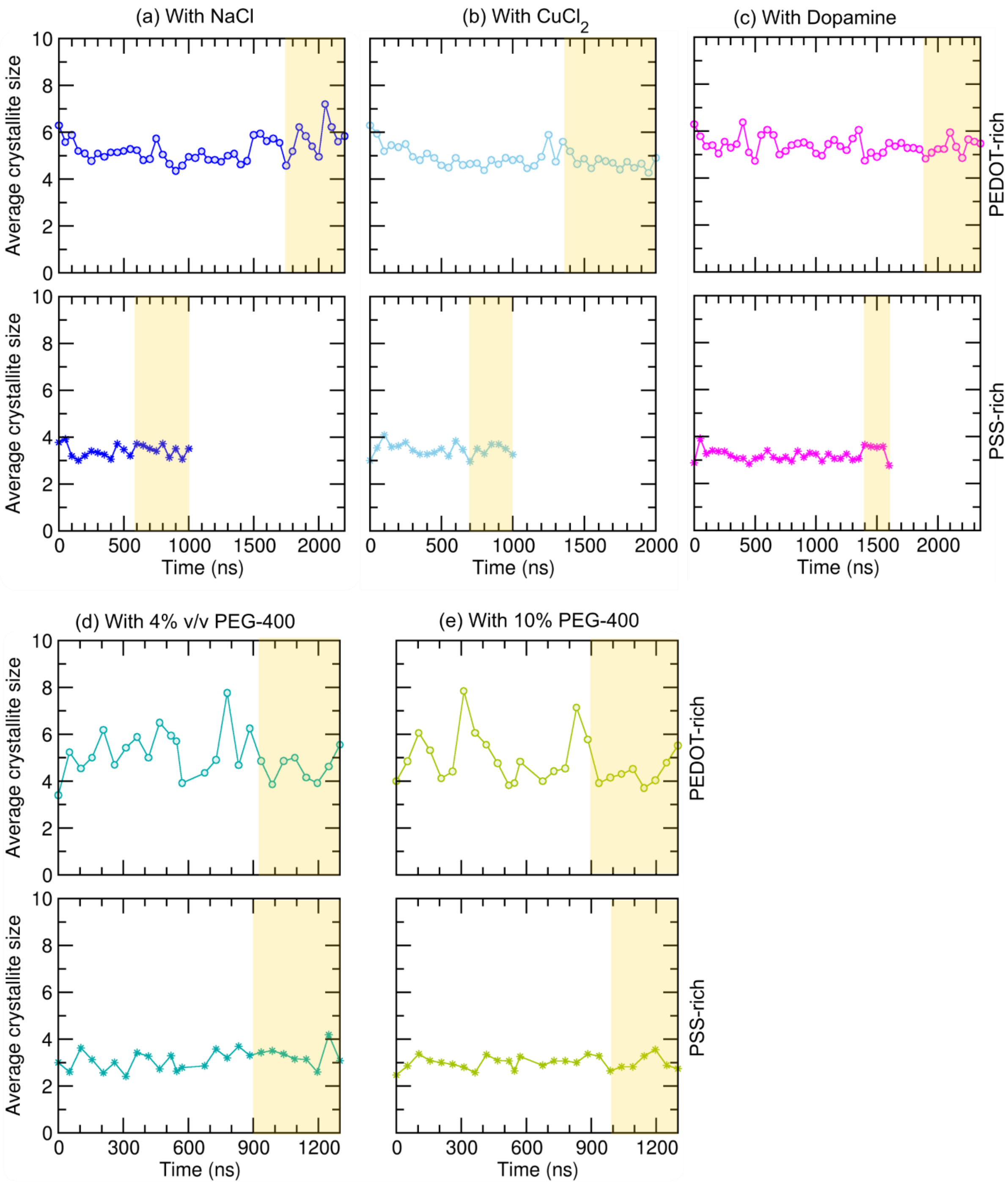

Figure S6. Average PEDOT lamella crystallite size as a function of simulation time for each chemical constituent. The shaded area indicates the equilibrated region. Under each chemical constituent, the top panels correspond to the PEDOT-rich phases, while the bottom panels show the corresponding PSS-rich phases.

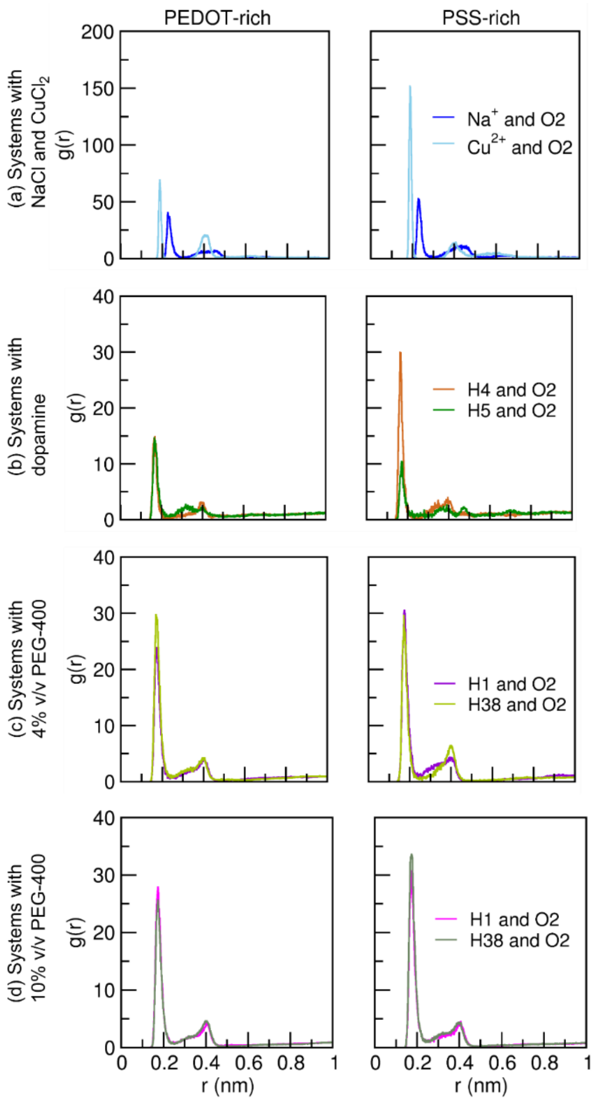

Figure S7. Radial distribution functions (RDFs) computed for systems exhibiting PEDOT-rich and PSS-rich phases, considering the chemical components examined in the study. (a) RDFs between Cu^{2+}/Na^{+} ions and an oxygen atom of the sulfonate (SO_3^-) group in the PSS chain (i.e., O2, as defined in the itp file[13]). (b) RDFs between the hydroxyl hydrogens of dopamine (H4 and H5, as defined in itp file[13]) and an oxygen atom of the sulfonate group in PSS. (c) RDFs between the terminal hydroxyl hydrogens of PEG-400 (H1 and H38, as defined in the itp file[13]) and the O2 oxygen atom of the sulfonate group in PSS. All RDFs were calculated from the final 50 ns of the NPT simulation trajectories.

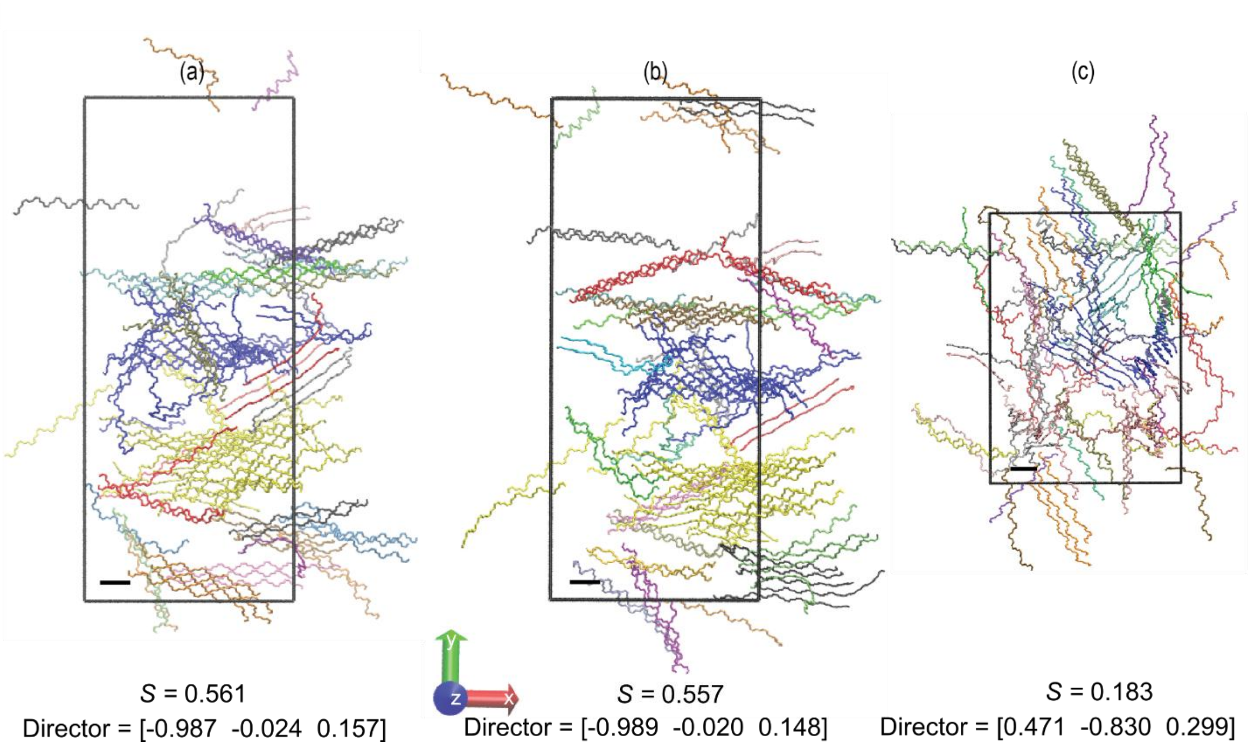

Figure S8. Arrangement of PEDOT chains in the PEDOT-rich phase with (a) NaCl, (b) dopamine and (c) PEG-400 (10% v/v). Only the carbon backbone of the PEDOT chain is shown, with each colour representing an individual lamella crystallite. The corresponding nematic order parameter and director are indicated at the bottom of each panel. Water, PSS and other molecular components are omitted for clarity. The scale bar represents 1 nm.

Table S5 – Radius of gyration and end-to-end distance of PEDOT chains in the absence of PEG and in PEG-containing PEDOT-rich and PSS-rich phases.

System	Rg (nm)	End-to-end distance (nm)
PEDOT-rich	1.3081 ± 0.0006	4.3450 ± 0.0033
PEDOT-rich + 4% v/v PEG-400	1.2898 ± 0.0006	4.2335 ± 0.0034
PEDOT-rich + 10% v/v PEG-400	1.2970 ± 0.0006	4.2638 ± 0.0036
PSS-rich	1.2542 ±0.0009	4.0360 ± 0.0057
PSS-rich + 4% v/v PEG-400	1.2725 ± 0.0009	4.1283 ± 0.0051
PSS-rich + 10% v/v PEG-400	1.2711 ± 0.0009	4.1771 ± 0.0047

The reported errors correspond to the standard deviation of the radius of gyration or end-to-end distance evaluated over the last 50 ns of each NPT simulation.

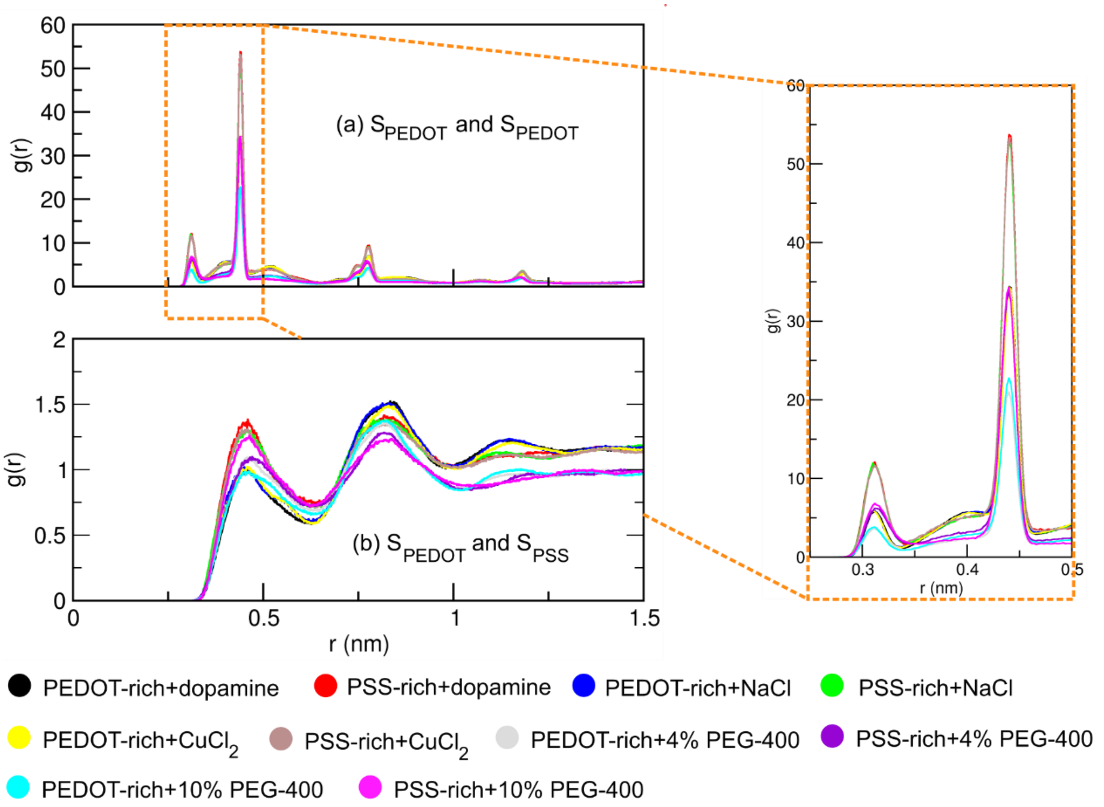

Figure S9. Radial distribution functions calculated for (a) sulfur atoms within PEDOT chains and (b) sulfur atoms between PEDOT and PSS chains. All RDFs were calculated from the final 50 ns of the NPT simulation trajectories. The right-hand panel provides an enlarged view highlighting the areas shown in Figures S9a.

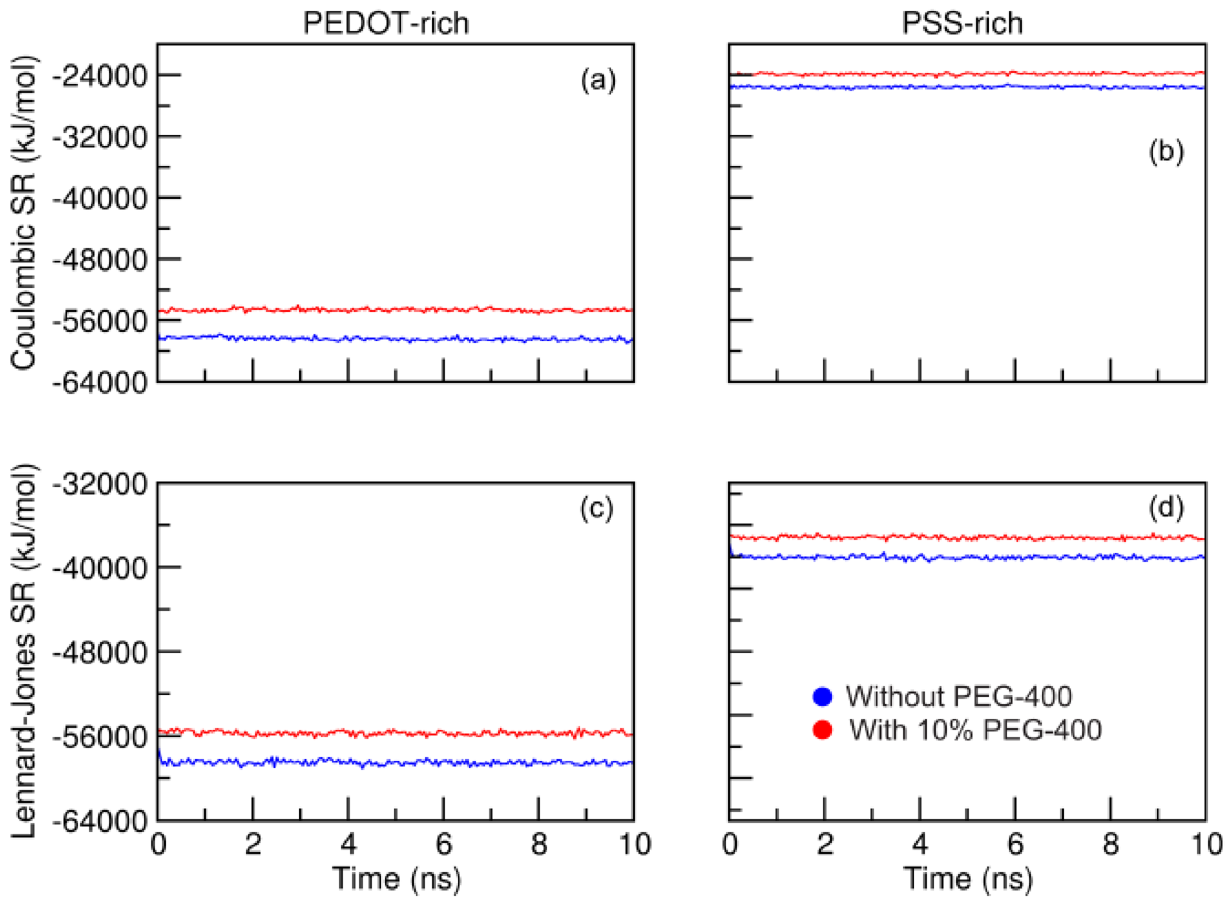

Figure S10. Coulombic short-range interaction energies between PEDOT and PSS in the (a) PEDOT-rich phase and (b) PSS-rich phases for systems with and without PEG-400, calculated at 300 K. Lennard-Jones short-range interaction energies between PEDOT and PSS in the (c) PEDOT-rich and (d) PSS-rich phases for systems with and without PEG-400, calculated at 300 K.

References

(1) Horta, B. A. C.; Merz, P. T.; Fuchs, P. F. J.; Dolenc, J.; Riniker, S.; Hünenberger, P. H. A GROMOS-Compatible Force Field for Small Organic Molecules in the Condensed Phase: The 2016H66 Parameter Set. *Journal of Chemical Theory and Computation* **2016**, *12* (8), 3825-3850. DOI: https://doi.org/10.1021/acs.jctc.6b00187.

(2) Jorgensen, W. L.; Maxwell, D. S.; Tirado-Rives, J. Development and Testing of the OPLS All-Atom Force Field on Conformational Energetics and Properties of Organic Liquids. *Journal of the American Chemical Society* **1996**, *118* (45), 11225-11236. DOI: https://doi.org/10.1021/ja9621760.

(3) Yabe, M.; Mori, K.; Ueda, K.; Takeda, M. Development of PolyParGen software to facilitate the determination of molecular dynamics simulation parameters for polymers. *Journal of Computer Chemistry, Japan-International Edition* **2019**, *5*, 2018-0034. DOI: https://doi.org/10.2477/jccjie.2018-0034.

(4) Frisch, M. J.; Trucks, G. W.; Schlegel, H. B.; Scuseria, G. E.; Robb, M. A.; Cheeseman, J. R.; Scalmani, G.; Barone, V.; Petersson, G. A.; Nakatsuji, H.; Li, X.; Caricato, M.; Marenich, A. V.; Bloino, J.; Janesko, B. G.; Gomperts, R.; Mennucci, B.; Hratchian, H. P.; Ortiz, J. V.; Izmaylov, A. F.; Sonnenberg, J. L.; Williams; Ding, F.; Lipparini, F.; Egidi, F.; Goings, J.; Peng, B.; Petrone, A.; Henderson, T.; Ranasinghe, D.; Zakrzewski, V. G.; Gao, J.; Rega, N.; Zheng, G.; Liang, W.; Hada, M.; Ehara, M.; Toyota, K.; Fukuda, R.; Hasegawa, J.; Ishida, M.; Nakajima, T.; Honda, Y.; Kitao, O.; Nakai, H.; Vreven, T.; Throssell, K.; Montgomery Jr., J. A.; Peralta, J. E.; Ogliaro, F.; Bearpark, M. J.; Heyd, J. J.; Brothers, E. N.; Kudin, K. N.; Staroverov, V. N.; Keith, T. A.; Kobayashi, R.; Normand, J.; Raghavachari, K.; Rendell, A. P.; Burant, J. C.; Iyengar, S. S.;

Tomasi, J.; Cossi, M.; Millam, J. M.; Klene, M.; Adamo, C.; Cammi, R.; Ochterski, J. W.; Martin, R. L.; Morokuma, K.; Farkas, O.; Foresman, J. B.; Fox, D. J. Gaussian 16 Rev. C.01. Wallingford, CT, 2016.

(5) Breneman, C. M.; Wiberg, K. B. Determining atom-centered monopoles from molecular electrostatic potentials. The need for high sampling density in formamide conformational analysis. *Journal of Computational Chemistry* **1990**, *11* (3), 361-373. DOI: https://doi.org/10.1002/jcc.540110311.

(6) De Oliveira, L. H.; Pinto, R. R.; Monteiro Filho, E. d. S.; Aznar, M. Density, refractive index, pH, and cloud point temperature measurements and thermal expansion coefficient calculation for PPG400, PE62, L64, L35, PEG400, PEG600, or PEG1000+ water systems. *Journal of Chemical & Engineering Data* **2021**, *66* (8), 2959-2975. DOI: https://doi.org/10.1021/acs.jced.0c01092.

(7) Lee, H.; de Vries, A. H.; Marrink, S.-J.; Pastor, R. W. A coarse-grained model for polyethylene oxide and polyethylene glycol: conformation and hydrodynamics. *The journal of physical chemistry B* **2009**, *113* (40), 13186-13194. DOI: https://doi.org/10.1021/jp9058966.

(8) Pronk, S.; Páll, S.; Schulz, R.; Larsson, P.; Bjelkmar, P.; Apostolov, R.; Shirts, M. R.; Smith, J. C.; Kasson, P. M.; Van Der Spoel, D.; Hess, B.; Lindahl, E. GROMACS 4.5: a high-throughput and highly parallel open source molecular simulation toolkit. *Bioinformatics* **2013**, *29* (7), 845-854. DOI: https://doi.org/10.1093/bioinformatics/btt055.

(9) Gerhardt, G.; Adams, R. N. Determination of diffusion coefficients by flow injection analysis. *Analytical Chemistry* **1982**, *54* (14), 2618-2620. DOI: https://doi.org/10.1021/ac00251a054.

(10) Buffle, J.; Zhang, Z.; Startchev, K. Metal flux and dynamic speciation at (bio) interfaces. Part I: Critical evaluation and compilation of physicochemical parameters for complexes with simple ligands and fulvic/humic substances. *Environmental science & technology* **2007**, *41* (22), 7609-7620. DOI: https://doi.org/10.1021/es070702p.

(11) Makki, H.; Troisi, A. Morphology of conducting polymer blends at the interface of conducting and insulating phases: insight from PEDOT:PSS atomistic simulations. *Journal of materials chemistry. C* **2022**, *10* (42), 16126-16137. DOI: https://doi.org/10.1039/D2TC03158B.

(12) Burgos-Mármol, J. J.; Patti, A. Molecular Dynamics of Janus Nanodimers Dispersed in Lamellar Phases of a Block Copolymer. *Polymers* **2021**, *13* (9), 1524.

(13) Guruge, A. G. PEDOT-PSS-Additives-Exposure, https://github.com/amaligg2024/PEDOT-PSS-Additives-Exposure/tree/main.
